# Parental Preconception Adversity and Offspring Health in African Americans: A Systematic Review of Intergenerational Studies

**DOI:** 10.1177/15248380221074320

**Published:** 2022-03-04

**Authors:** Josiah A. Sweeting, Adebisi A. Akinyemi, Ellen Alison Holman

**Affiliations:** 1 Department of Psychological Science, 8788 University of California, Irvine, CA, USA; 2 Sue & Bill Gross School of Nursing, 8788 University of California, Irvine, CA, USA

**Keywords:** intergenerational, historical trauma, stress, preconception adversity, health disparities, African American

## Abstract

**Background:** This systematic review explores the empirical literature addressing the association between parental preconception adversity and offspring physical health in African-American families. **Method**: We conducted a literature search in PubMed, Web of Science, PsycINFO, CINAHL, and Scopus through June 2021. Articles were included if they: reported data about at least two generations of African-American participants from the same family; measured parental preconception adversity at the individual level; measured at least one offspring physical health outcome; and examined associations between parental adversity and child health. **Results**: We identified 701 unique articles; thirty-eight articles representing 30 independent studies met inclusion criteria. Twenty-five studies (83%) reported that parental preconception adversity was associated with child health; six studies (20%) reported that parental preconception adversity was not associated with at least one offspring outcome; several studies reported both. Only six studies (20%) reported an association specific to African Americans. **Conclusion**: Empirical evidence linking parental preconception adversity with offspring physical health in African Americans is limited and mixed. In the current literature, very few studies report evidence addressing intergenerational associations between parental preconception adversity and offspring physical health in the African-American population, specifically, and even fewer investigate forms of parental preconception adversity that have been shown to disproportionately affect African Americans (e.g., racism). To better understand root causes of racial health disparities, more rigorous systematic research is needed to address how intergenerational transmission of historical and ongoing race-based trauma may impact offspring health among African Americans.

African Americans (AAs) are more likely than whites to experience poor health throughout the lifespan (Carnethon et al., 2017; Mehta et al., 2013). Historical trauma theory (Sotero, 2006) suggests that this is due to the unique history of race-based adversity experienced by AAs: slavery, economic marginalization, ongoing systemic violence, and discrimination. AAs also experience increased prevalence of adversity that is common across all races (e.g., domestic violence; Boardman & Alexander, 2011; Roberts et al., 2011), suggesting that AAs experience multiple forms of significant adversity (i.e., stress, trauma) with effects that may have rippled across generations and contributed to the widespread health inequalities seen today.

Historical trauma theory (Sotero, 2006) posits that affected groups experience physical, psychological, and economic disparities that persist across generations. These disparities also contribute to AAs being at greater risk for adversities experienced across all races, such as adverse childhood experiences (ACEs), environmental exposures, and stress across several domains (e.g., financial, relationship; Boardman & Alexander, 2011; Roberts et al., 2011). Moreover, race-specific adversity (e.g., institutional racism, interpersonal discrimination) permeates multiple domains of life for AA families (DeGue et al., 2016; Williams & Collins, 2001). Importantly, these experiences have been linked to several negative physiological consequences and physical health outcomes.

For populations experiencing historical trauma, research suggests that the adversity they disproportionately experience (e.g., ACEs, discrimination, low socioeconomic status, SES; Pager & Western, 2012; Sacks & Murphey, 2018) is more likely to result in epigenetic alterations (Conching & Thayer, 2019) that can affect gene expression and produce biological dysfunction. Such changes have been identified in several domains including the immune (Dhabhar, 2014), neuroendocrine (Marsland et al., 2017), and cardiovascular (Hill et al., 2017) systems, epigenetic aging (Brody et al., 2016), and the methylation of genes involved in immune responses and threat-related amygdala reactivity (Houtepen et al., 2016). Specific examples include exposure to racism and discrimination being associated with lower parasympathetic cardiac modulation as measured by heart-rate variability (HRV; Hill et al., 2017) and several other indicators of poor health (Lewis et al., 2015). These physiologic correlates of discrimination and racism likely increase risk for cardiovascular disease (Barber et al., 2016) and other chronic health problems (Mouzon et al., 2017). Beyond negatively impacting individuals directly exposed to adversity, a growing body of empirical work has illustrated how these health consequences can also be observed across generations and how they may occur.

## Understanding Intergenerational Transmission

Several mechanisms are thought to link adversity experienced in one generation with a future generation’s physical health (Choi et al., 2017). Investigators have mainly explored pregnant mothers and how negative exposures *during the prenatal period* are associated with increased risk of poor offspring health (Thayer & Kuzawa, 2011). The developmental origins of health and disease (DOHaD) hypothesis describes a period of great epigenetic elasticity during fetal development occurring simultaneously with the transfer of hormones and other information between the mother and child (Kuzawa & Quinn, 2009). Consequently, the intrauterine environment plays an instrumental role in shaping the offspring epigenome. Maternal mood and stress during pregnancy are associated with DNA methylation in offspring tissues which is associated with greater offspring central adiposity and body mass index (Cao-Lei et al., 2015) among several other negative health outcomes. Ultimately, this work suggests that prenatal maternal adversity can cause harmful epigenetic patterns in offspring through intrauterine signaling with serious long-term health repercussions.

Two other research literatures also address potential mechanisms by which maternal adversity experienced *before pregnancy* (henceforth preconception) may contribute to behaviors that impact the health and epigenome of future generations. In the first, early-life stress (e.g., ACEs) is associated with greater risk of early pregnancy during adolescence (Madigan et al., 2014); in the second, teen pregnancies are linked to increased risk of intrauterine growth restriction (Malabarey et al., 2012), low birth weight (LBW), and preterm birth (PTB; Torvie et al., 2015). Importantly, these neonatal outcomes have implications for subsequent offspring physical health, including greater body fat percentage and insulin resistance (Crume et al., 2014) and metabolic syndrome (Parkinson et al., 2013). However, direct associations between parental ACEs in one generation and physical health outcomes in subsequent generations are infrequently studied and focus almost exclusively on maternal, as opposed to paternal, adversity. Consequently, limited work has explored across generations to determine whether parental preconception adversity is directly linked to children’s health, with even less work accounting for how fathers’ adversity experiences may play a role in this potential link.

## Intergenerational Transmission of Historical Trauma and Health

The intergenerational health consequences of historical trauma experienced by specific populations have been studied primarily among Holocaust survivors and Indigenous populations. Holocaust survivors’ children often experience reduced cortisol excretion, lower overall cortisol levels (Bierer et al., 2014), and changes in DNA methylation of stress regulatory genes (Yehuda et al., 2016). For Indigenous populations, studies have highlighted the intergenerational impact of Indian Residential Schools documenting that children from families with at least one parent or grandparent attendee report poorer self-rated health and higher rates of chronic and infectious diseases (Wilk et al., 2017). When it comes to exploring similar issues in the AA community, empirical work has shown links between several forms of exposure to racism and adverse offspring outcomes (Bower et al., 2018; Dominguez, 2011; Slaughter-Acey et al., 2016), but overwhelmingly focuses on prenatal exposure to these specific forms of adversity. As a result, there is a need to examine closely the evidence for intergenerational health associations with respect to distinct experiences of historical trauma (e.g., discrimination and racism) in this population prior to conception.

### Overview of the Present Review

Research has documented that *prenatal* maternal stress is associated with offspring health, and that parental preconception adversity has potential behavioral repercussions (e.g., teen pregnancy), which may have consequences for offspring physical health. However, researchers less often explore direct links between parental *preconception* adversity and their *offspring’s* physical health, especially in AAs. Furthermore, while recent research has explored the intergenerational health impacts of historical and ongoing adversity in Holocaust survivors and Indigenous populations, less empirical work addresses how the unique, preconception adversity experiences of the AA population may affect their offspring’s physical health outcomes across generations. Consequently, this review examines this literature with the goal of providing a synopsis and potential roadmap for future work in this important area of research.

## Method

We conducted a computerized, systematic search of five electronic databases (CINAHL, PsycINFO, PubMed, Scopus, Web of Science) through June 2021 to identify empirical studies addressing the intergenerational links between parental preconception adversity and offspring physical health outcomes. This review is registered in PROSPERO under protocol CRD42018105369. Studies consistent with the following inclusion criteria were reviewed:

1. Reports data from AAs living in the U.S.
2. Includes participants from at least 2 separate generations of the same family (e.g., mother/father, daughter/son)
3. Measures at least 1 form of parental adversity that:
  a. Is measured at the individual level for the parent and not reported by the offspring
  b. Occurred prior to the conception of the specific offspring in the study
4. Includes a measure of at least 1 physical health outcome in the offspring gathered via independent information sources (e.g., medical records), offspring self-report, or parent report
5. Examines the association between parental preconception adversity and the index child’s physical health outcome.

### Justification for Inclusion Criteria

Inclusion criteria were partially established through the identification of several related systematic reviews (Alhusen et al., 2016; Gone et al., 2019), but were further adapted to address the specific aims of the current review. Due to the unique historical and ongoing adverse experiences of AAs in the U.S. (Alexander, 2010; Anderson, 2016), this review included only studies focusing on individuals and families residing in the U.S. Given the research documenting the offspring health consequences of historical trauma in Indigenous populations and Holocaust survivors (e.g., depressive symptoms, epigenetic changes; Walls & Whitbeck, 2012; Yehuda et al., 2016), and the historical experience of AAs in the U.S., examining similar intergenerational processes in AAs is needed. Furthermore, we only included studies reporting data from at least two separate generations of AAs from the same family as this is essential for exploring the intergenerational effects of parental adversity on offspring physical health. Studies must have clearly assessed parental *preconception* adversity to understand the intergenerational health impacts of parental adversity beyond what has already been established in the prenatal stress literature.

We included studies examining only individual-level parental adversity due to the difficulty in gauging the direct impact of neighborhood-level experiences on individuals and their families, and its possible confounding the link between parental adversity and child health. Additionally, we only included studies that captured adversity exposure directly reported by the parent; studies in which offspring reported on their parent’s adverse experiences were omitted due to concerns about the accuracy of these accounts as offspring may not be fully aware of their parents’ lifetime exposures. Finally, we included studies reporting at least one measure of offspring physical health with a particular focus on those that captured these outcomes through independent information sources (e.g., medical charts) and offspring self-report as these present the most objective and least biased measures. Although parent report of offspring physical health is subject to considerable bias as parents may be reluctant to disclose their offspring’s physical health status candidly or may unknowingly report health issues incorrectly, we also included these studies in the review to capture how they compare to studies using independent and less biased measures.

### Procedure

**Figure 1.**
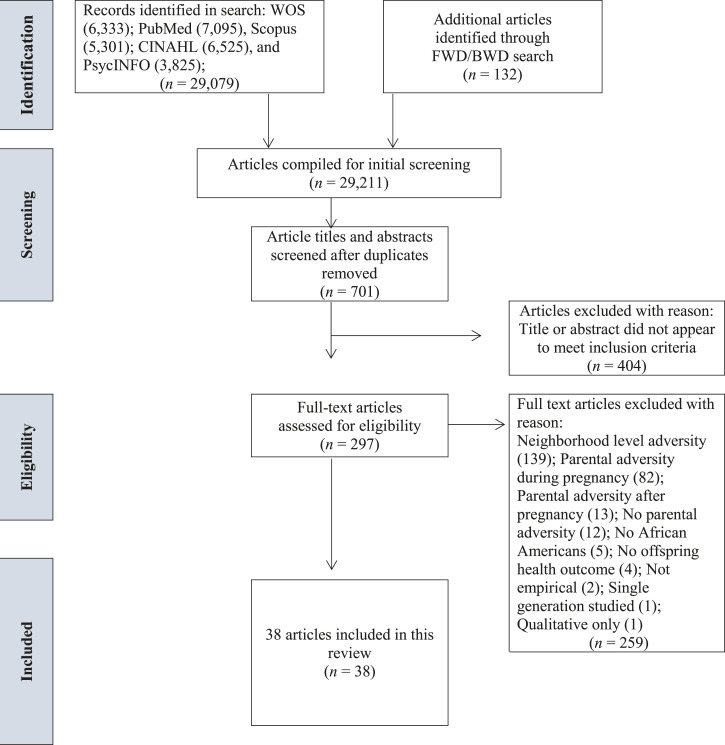
Flow diagram for article selection.

Keyword, controlled vocabulary, or MeSH term combinations were constructed to represent each component of the review topic (see Supplemental Appendix A). Searches were restricted to English-language journal articles and dissertations. Two independent reviewers (J.S. and A.A.) conducted all searches separately and performed an initial screening of articles by title. Next, the titles and abstracts of relevant articles were reviewed and those appearing to meet inclusion criteria were further assessed for eligibility by examining the full text of the article. All results were compared at each step and any discrepancies were resolved by the two independent reviewers (J.S. and A.A.) and an advisor (E.A.H.) through a consensual, iterative process. The two coders agreed on 78% and negotiated 22% of the articles when reviewing article titles. After reviewing article abstracts, the coders agreed on 89% and negotiated 11% of the articles for full review. Following full-text review, coders demonstrated 95% agreement and negotiated 5% of the final collection of articles. The two independent reviewers also conducted a forward and backward search of the included articles (i.e., they screened articles that were cited by or cited these articles) to identify and add any additional articles meeting inclusion criteria to the final collection (see Figure 1). Lastly, the grey literature was assessed using list-servs of American Psychological Association’s Division 38 (Society for Health Psychology) and 56 (Trauma Psychology), as well as the International Society for Traumatic Stress Studies’ Intergenerational Transmission of Trauma and Resilience special interest group , asking for any relevant studies that met inclusion criteria.

### Data Synthesis

Two authors (J.S. and A.A.) reviewed all articles that met the full inclusion criteria and extracted data to create a table of evidence (see Supplemental Appendixes B-E). All authors then reviewed and discussed findings to identify patterns in associations reported between parental preconception adversity and child physical health. A.A. and J.S. also conducted quality assessments of all included articles using the Newcastle-Ottawa Scale (NOS; Wells et al., 2000; see Supplemental Appendices F, H, J).

### Quality Assessment

A.A. and J.S. conducted quality assessments of each article from the 30 studies reviewed using the NOS (Wells et al., 2000; see Supplemental Appendixes F-K). Most studies (*n* = 23, 77%) were assessed with an adapted version of the NOS for cohort studies based on Kansagara et al. (2017); six studies (20%) were analyzed with a NOS adaptation for cross-sectional studies based on Herzog et al. (2013) while one study was assessed using an adaptation for case-control studies. A large majority of studies reviewed (*n* = 27, 90%) were classified as having either a moderate or high risk of bias for several reasons including having inadequate or incomplete participant response rate information, non-representative or small samples, retrospective measures of parental preconception adversity, parental report of offspring health, and not accounting for important confounders (e.g., prenatal adversity, current stress levels, offspring exposure to adversity, race/ethnicity). As a result, the quality of the current studies significantly limits our ability to provide a comprehensive assessment of the association between parental preconception adversity and offspring health in AA families.

## Results

### Search Results

Results from the three-step process used to determine eligibility for inclusion is depicted in Figure 1. The initial search results returned 6333 articles in Web of Science (WOS), 7095 articles in PubMed, 6525 articles in CINAHL, 5301 articles in Scopus, 3825 articles in PsycINFO, and 132 articles identified through the backward and forward search for a total of 29,211 results. After deleting duplicates, 701 articles remained; after reviewing article titles and abstracts, 404 articles were dropped because the titles did not mention relevant topics or the abstracts did not appear to meet criteria. Next, the full texts of the remaining 297 articles were assessed for eligibility. Of these articles, 259 were excluded because: parental adversity was assessed at the neighborhood level (*n* = 139), not explicitly measured during the preconception period (e.g., during pregnancy; *n* = 82), or after pregnancy (*n* = 13); no parental adversity was measured (*n* = 12); the sample did not include AAs or did not give the percentage of the sample that was AA (*n* = 5); no offspring physical health outcome was reported (*n* = 4); the study was not empirical (*n* = 2); only a single generation was studied (*n* = 1); and results were qualitative (*n* = 1). Ultimately, 38 articles representing 30 unique studies were included in the review.

### Study Characteristics

Supplemental Appendices B-E present the characteristics and key findings of the 38 papers from these 30 studies. Each appendix covers one of four categories: studies with entirely AA study samples (*n* = 5; see Supplemental Appendix B); studies with partial AA samples that examine the role of race in the association between parental preconception adversity and offspring physical health (*n* = 5; see Supplemental Appendix C); studies with partial AA samples that *do not* examine the role of race in the association between parental preconception adversity and offspring physical health (*n* = 10; see Supplemental Appendix D); and studies with parent-reported offspring health outcomes (*n* = 10; see Supplemental Appendix E). Appendices B,C,D include studies with non-parental, independent report of offspring health. Most of the studies were published after 2010 (*n* = 25, 83%); most studies used a cohort design (*n* = 24, 80%); and six studies used a single group cross-sectional design (*n* = 6, 20%). Nineteen studies used retrospective (63%), and 11 used prospective (37%) approaches. It is also important to note that three separate studies produced eleven articles, resulting in more than 30 total entries in the supplemental appendices.

### Sample Characteristics

Sample sizes ranged greatly with the smallest including 31 participants and the largest including 9350; the median was 493. Three studies produced eleven articles that were included in this review; one study produced five articles (Cheng et al., 2016; Witt et al., 2014a, 2014b, 2015, 2016), one published four articles (Cammack et al., 2019; Flagg et al., 2014; Ihongbe 2018; Strutz et al., 2014), a third study yielded two articles (Brunst et al., 2017; Sternthal et al., 2011). This left 30 unique study samples, six of which (20%) included only AAs, six (20%) had >50% AA participants, and the remaining 18 (60%) had <50% AA respondents. Most of the 30 unique studies used convenience samples (*n* = 25, 83%), two had clinical samples (7%), and three utilized nationally representative samples (10%).

### Parental Adversity Measures

All 30 studies focused on *maternal* adversity. Most studies measured maternal childhood adversity (*n* = 21, 70%) or general lifetime adversity (*n* = 4, 13%); two studies reported both in separate articles; three other studies (10%) explored race-specific adversity. Of the 21 studies measuring childhood adversity, ten (48%) captured ACEs in general; four (19%) focused on childhood SES; three (14%) focused specifically on childhood abuse; two (10%) assessed general childhood stress (e.g., assault, loss, physical danger); one (5%) measured early-life neighborhood conditions (e.g., disorder, social control, violence); and another one (5%) measured both ACEs and childhood SES. Four studies (19%) included general lifetime adversity measures (e.g., bereavement, economic strain, adulthood abuse, relationship problems) and traumatic events (e.g., disasters, interpersonal trauma). Two of these studies (10%) reported both childhood adversity (e.g., abuse, early-life neighborhood conditions, SES; Cammack et al., 2019; Sternthal et al., 2011) and general lifetime adversity (e.g., stressful events, trauma; Brunst et al., 2017; Strutz et al., 2014). Lastly, three studies (14%) measured race-specific adversity including exposure to several forms of racism and racial discrimination in childhood and adolescence (e.g., direct, indirect, vicarious).

### Offspring Health Outcomes

Eighteen studies (60%) included independent reports of offspring health (e.g., biological data, medical records, and offspring report) exclusively while nine studies (30%) only included parent-reported offspring health measures; three studies (10%) included both independent and parent-reported offspring health. Most studies reported health outcomes that were captured at birth (*n* = 24, 80%) while two (7%) measured outcomes at 4 months of age; the remaining four studies’ (13%) outcomes were measured between birth and seventeen years of age. Because many studies reported more than one offspring health outcome (*n* = 12, 40%), the outcome numbers reported below may not add up to exactly 30. The most common health outcomes measured were infant birth weight (*n* = 13, 43%), birth timing or gestational age (*n* = 10, 33%), and premature or PTB status (*n* = 9, 30%). Other infant-specific health outcomes included stillbirth (*n* = 3, 10%), fetal growth measures (*n* = 2, 7%), respiratory sinus arrhythmia (RSA; *n* = 2, 7%), and miscarriage (*n* = 2, 7%). Admission to special care nursery and the length of hospital stay were each captured only once across the studies. Finally, several child health outcomes related to asthma (e.g., control, cytokine production, diagnosis; *n* = 3, 10%), cord blood immunoglobulin E (IgE) levels (*n* = 1, 3%), obesity status (*n* = 1, 3%), overall health status (*n* = 1, 3%), startle response (*n* = 1, 3%), and HRV (*n* = 1, 3%) were measured.

### Parental Adversity and Independently Reported Offspring Health

#### Studies Using 100% AA Samples

Table 1 reflects a brief tally of all results of the review; Supplemental Appendix B provides a detailed summary of the five studies that had 100% AA samples and compared independently-reported health of children whose mothers reported preconception adversity to children whose mothers did not. Four studies (80%) captured offspring birth outcomes (e.g., birth timing, birth weight, fetal growth) and one study (20%) explored adolescent outcomes (e.g., child HRV, startle response; see Supplemental Appendix B). Parental preconception adversity was significantly associated with poor offspring physical health in four of these studies. Specifically, maternal early-life adversity (e.g., cumulative stress, neighborhood disorder) was significantly associated with birth timing in two studies (40%; Gillespie et al., 2017; Sealy-Jefferson et al., 2019); maternal childhood abuse (e.g., emotional and physical) was significantly associated with heightened offspring startle response and HRV ratio (20%; Jovanovic et al., 2011), a physiologic measure previously linked to greater cardiovascular disease risk and all-cause mortality (Fang et al., 2020). Another study (20%) linked *indirect* maternal exposure to racism in childhood with offspring LBW (Hilmert et al., 2014).

**Table 1.** Results from Studies Addressing Intergenerational Transmission of Adversity in African-American Families.

Author	Maternal preconception adversity associated with child health?		Racial differences identified in association between maternal preconception adversity and child health?	
**Studies using all ** **African-American** ** samples**				
Gillespie et al	YES		NA	
Hilmert et al	YES		NA	
Jovanovic et al	YES		NA	
Rowell	NO		NA	
Sealy-Jefferson et al	YES		NA	
Total for all AA Samples	YES = 4	NO = 1		
**Studies testing racial differences in link between parental preconception adversity and child health**				
Dominguez et al	YES		YES	
Gray et al	YES		NO	
Margerison-Zilko et al	YES		NO	
Masho et al	NO		NO	
Seng et al	NO		NO	
Total for testing racial differences	YES = 3	NO = 2	YES = 1	NO = 4
**Studies not testing racial differences in link between parental preconception adversity and child health**				
Blackmore et al	YES		NA	
Chen et al	YES		NA	
Cheng et al^a^	YES		NA	
Cowell et al	NO		NA	
Freedman et al	YES		NA	
Jones et al	YES		NA	
Mersky et al	YES		NA	
Miller et al	YES		NA	
Noll et al	YES		NA	
Smith et al	YES		NA	
Sternthal et al^c^	YES		NA	
Witt et al 2014a^a^	YES		NA	
Witt et al 2014b^a^	YES		NA	
Witt et al 2015^a^	YES		NA	
Witt et al 2016^a^	YES		NA	
Total for not testing racial differences	YES = 14	NO = 1		
**Studies with ** **parent-reported** ** offspring health**				
Astone et al	YES		NA	
Brunst et al^c^	YES		NA	
Cammack et al^b^	YES		NO	
Daniels et al	YES		NA	
Flagg et al^b^	NO		NO	
Freeman et al	NO		NO	
Gavin et al	YES		NO	
Hillis et al	YES		NO	
Ihongbe^b^	YES		NO	
Kerkar et al	YES		NO	
Lê-Scherban et al	YES		NO	
Stein et al	YES		NO	
Strutz et al^b^	YES		NO	
Total for parent-reported offspring health	YES = 11	NO = 2	YES = 0	NO = 10
Grand total	YES = 32	NO = 6	YES = 1	NO = 14

Table note. NA refers to not applicable.

^a^These five papers report data from the same study.

^b^These four papers report data from the same study.

^c^These two papers report data from the same study.

However, four of these studies (80%) also reported non-significant associations between maternal preconception adversity and offspring physical health in AA families (Hilmert et al., 2014; Jovanovic et al., 2011; Rowell, 2020; Sealy-Jefferson et al., 2019). General, maternal childhood adversity (e.g., cumulative ACEs, neighborhood disorder, physical and sexual abuse) was not associated with several birth or early-life offspring outcomes (e.g., birth weight, gestational age; Jovanovic et al., 2011; Rowell, 2020; Sealy-Jefferson et al., 2019), and *direct* maternal exposure to racism in childhood was not associated with fetal growth (Hilmert et al., 2014). Thus, of the five studies with all AA samples, the results were mixed and inconclusive; four reported that parental adversity was associated with some offspring health outcomes, but four also reported some non-significant findings.

#### Studies Testing for Racial Differences

Five studies that had partial AA samples tested for racial differences in the association between parental preconception adversity and offspring health (see Supplemental Appendix C). Four of these studies (80%) measured offspring birth outcomes (e.g., birth timing, birth weight, gestational age) and one study (20%) explored early-life outcomes (e.g., infant RSA). In two studies (40%), maternal childhood adversity (e.g., ACEs, abuse, or violence) was associated with PTB (Margerison-Zilko et al., 2017) and infant RSA (Gray et al., 2017)—an index of parasympathetic nervous system activity (Beauchaine, 2001) that heightens risk for chronic disease (Masi et al., 2007)—but no differences were found between AA and white mothers in either study. Another study (20%) reported that vicarious maternal exposure to racism in childhood was significantly associated with offspring birth outcomes in AA families, but not in white families (Dominguez et al., 2008).

Four of these studies (80%) also reported non-significant associations between preconception adversity and offspring physical health (Dominguez et al., 2008; Margerison-Zilko et al., 2017; Masho et al., 2015; Seng et al., 2011). In Dominguez et al., (2008), *direct* maternal exposure to racism in childhood was not linked to offspring birth weight, and three studies found no association between economic strain, loss, child maltreatment, or substance use and offspring birth outcomes in any racial group (Margerison-Zilko et al., 2017; Masho et al., 2015; Seng et al., 2011). Thus, of five studies addressing racial differences in the association between maternal preconception adversity and child health, three studies (60%) reported significant associations, but only one (20%) of them documented a stronger association in AAs than whites, while four studies also reported non-significant race-specific findings (see Table 1).

#### Studies Not Testing Racial Differences

Fifteen articles, representing 11 unique studies, used partial AA samples without testing for racial differences in the association between parental preconception adversity and independently reported offspring health (see Supplemental Appendix D). Most studies (*n* = 8, 73%) captured offspring birth outcomes (e.g., admission to special care nursery, birth timing, birth weight, fetal death, fetal growth, length of hospital stay, PTB status) while two (18%) explored early-life outcomes (e.g., cord blood IgE levels, infant RSA) and one (9%) measured adolescent outcomes (e.g., asthma control, cytokine production). Ten of the 11 studies (91%) reported at least one significant association between preconception maternal adversity and offspring physical health outcomes (Blackmore et al., 2016; Chen et al., 2017; Cheng et al., 2016; Freedman et al., 2017; Jones et al., 2019; Mersky & Lee, 2019; Miller et al., 2017; Noll et al., 2007; Smith et al., 2016; Sternthal et al., 2011; Witt et al., 2014a, 2014b, 2015, 2016). Six of these (55%) examined maternal childhood adversity (e.g., ACEs, sexual abuse) and identified significant links with birth and other early-life outcomes (Blackmore et al., 2016; Freedman et al., 2017; Jones et al., 2019; Mersky & Lee, 2019; Noll et al., 2007; Smith et al., 2016).

Three studies (27%; Chen et al., 2017; Miller et al., 2017; Sternthal et al., 2011) reported significant associations between maternal early life disadvantage (e.g., low childhood SES, childhood family economic hardship) and birth outcomes (Miller et al., 2017) and other early-life and adolescent outcomes (Chen et al., 2017; Sternthal et al., 2011). One study (9%; represented in five articles)—the Early Childhood Longitudinal Study-Birth Cohort—used a nationally representative sample of 9350 mother–child dyads, and reported significant associations between maternal preconception stressful life events (PSLEs; e.g., bereavement, divorce) and birth outcomes such as very LBW (Cheng et al., 2016; Witt et al., 2014a, 2015, 2016) and PTB (Witt et al., 2014b), but not LBW (Witt et al., 2014a). Finally, one study (9%) reported no significant link between maternal ACE exposure and infant birth timing (Cowell et al., 2021). In sum, ten independent studies with multiracial samples reported significant links between maternal preconception adversity and offspring physical health, but did not examine racial differences in the strength of these associations while one study reported a non-significant finding (see Table 1).

### Parental Adversity and Parent-Reported Offspring Health

Thirteen articles, representing ten unique studies, examined associations between maternal preconception adversity and parent-reported offspring health outcomes (see Supplemental Appendix E). Of these ten studies, six (60%) had <50% AA respondents, three (30%) had >50% AA participants, and only one included 100% AAs. Most studies (*n* = 8, 80%) measured offspring birth outcomes (e.g., birth timing, birth weight, fetal death) while the remaining two (20%) explored early life outcomes (e.g., asthma diagnosis, obesity status, overall health status). All but one study (*n* = 9, 90%) reported at least one significant association between preconception maternal adversity and poor offspring physical health (Astone et al., 2007; Brunst et al., 2017; Cammack et al., 2019; Daniels et al., 2020; Gavin et al., 2011; Hillis et al., 2004; Kerkar et al., 2021; Lê-Scherban et al., 2018; Stein et al., 2000). Seven studies (70%) examined maternal childhood adversity (e.g., ACEs, neighborhood social control and disorder, SES), with six identifying at least one significant association with birth outcomes (e.g., timing, weight, fetal death; Astone et al., 2007; Gavin et al., 2011; Hillis et al., 2004; Kerkar et al., 2021; Stein et al., 2000) and other early-life outcomes (e.g., asthma diagnosis, obesity status, overall health; Lê-Scherban et al., 2018). One study reported that AA mothers exposed to vicarious childhood (≤age 12) racial discrimination and direct adolescent (ages 13–19) racial discrimination had significantly higher PTB risk than AA mothers who were not exposed to such discrimination (Daniels et al., 2020).

Three articles (Cammack et al., 2019; Flagg et al., 2014; Strutz et al., 2014) and one dissertation (Ihongbe, 2018) reported data from the same National Longitudinal Study of Adolescent to Adult Health (“Add Health”), a large nationally representative sample comprised of over 90,000 adolescents. Findings from this study were mixed, suggesting that while maternal preconception adversity (e.g., childhood abuse, chronic stressors, neighborhood violence exposure) was significantly associated with birth outcomes (e.g., birth weight, PTB, very LBW; Cammack et al., 2019; Ihongbe, 2018; Strutz et al., 2014), grandparental exposure to neighborhood disorder was not associated with their grandchild’s birth weight (Flagg et al., 2014). Finally, Freeman et al. (2014) found no significant link between maternal early life poverty and risk of infant LBW. To summarize, ten independent studies investigated associations between maternal preconception adversity and parent-reported offspring physical health outcomes with all but one study reporting significant findings (see Table 1).

### Mechanisms for Intergenerational Transmission of Adversity

Only seven studies (23%) identified and measured potential mechanisms linking parental preconception adversity with offspring health. Most studies explored how maternal preconception adversity affected various prenatal physiological processes including changes to immune function and inflammation, cortisol levels, hemodynamic factors related to blood pressure (BP), and placental tissue telomere length (TL). In Miller et al. (2017), a panel of maternal inflammatory biomarkers was investigated (interferon-γ; interleukins, or IL-6, 8, 10, and 13; tumor necrosis factor-α), and IL-6 levels mediated links between maternal childhood disadvantage and several infant outcomes including birth weight, PTB, small for gestational age, length of hospital stay, and admission to special care nursery. Gillespie et al. (2017) showed that maternal cortisol mediated the association between a mother’s childhood stress and her offspring’s birth timing, but only in women giving birth after spontaneous labor. In contrast, Noll et al. (2007) found that maternal cortisol did not mediate the association between the mother’s childhood sexual abuse and her baby’s PTB status. Hilmert et al. (2014) reported that greater maternal exposure to indirect racism in childhood interacted with prenatal increases in diastolic BP (DBP) to predict lower infant birth weight. Finally, Jones et al. (2019) demonstrated that placental tissue TL moderated the association between a mother’s ACE exposure and infant stress responsivity.

Maternal preconception adversity also demonstrated associations with behavioral and lifestyle factors that have been previously linked to adverse outcomes for newborns. In Smith et al. (2016), prenatal smoking and substance use accounted for most of the differential impact of maternal ACE exposure on infant birth weight; prenatal smoking was also the strongest mediator of the link between maternal ACEs and her infant’s gestational age. Similarly, maternal childhood maltreatment (e.g., childhood sexual abuse) was linked to adolescent substance use and prenatal tobacco and alcohol use, ultimately affecting infant birth weight (Gavin et al., 2011) and PTB status (Noll et al., 2007). Prenatal alcohol use also partially mediated the link between maternal childhood sexual abuse and PTB status (Noll et al., 2007).

## Discussion

The literature reviewed provides mixed and inconclusive evidence about the association between maternal preconception adversity and offspring physical health in AA families (see Table 2 and Supplemental Appendices B–E). We reviewed 38 articles, representing 30 unique studies; 25 (83%) of these studies documented that maternal preconception adversity was associated with poor health outcomes in their offspring (e.g., LBW, PTB, RSA). Six (20%) also reported at least one non-significant association, with some studies reporting both. This literature suggests that several types of maternal preconception adversity (e.g., ACEs, overall lifetime adversity, neighborhood disadvantage) may impact a range of birth and early life offspring physical health outcomes in diverse samples. However, findings specifically addressing whether these associations are stronger in AA samples were both limited and quite mixed. Five of the 25 studies reporting a significant association between preconception adversity and poor child health found this association was more likely in AAs who experienced preconception adversity than in AAs who did not (Daniels et al., 2020; Gillespie et al., 2017; Hilmert et al., 2014; Jovanovic et al., 2011; Sealy-Jefferson et al., 2019), one documented a stronger association in AAs than in whites (Dominguez et al., 2008), two found no role for race in the strength of this association (Gray et al., 2017; Margerison-Zilko et al., 2017), and 17 studies did not examine racial differences (See Supplemental Appendixes D & E). Similarly, preconception exposure to racism in AA moms was also shown to significantly impact offspring health, but these links appear dependent on the type (e.g., direct, indirect/vicarious racism) and timing (e.g., childhood vs. adulthood) of exposure (Dominguez et al., 2008; Hilmert et al., 2014). Thus, while the literature generally suggests that preconception maternal adversity is a risk factor for poor offspring health across demographically diverse samples, the heterogenous nature of adversity, outcome, and control variable assessments, and the different types of analyses conducted in these studies prevent us from drawing firm conclusions about this intergenerational health risk in AA families.

**Table 2.** Summary Table of Critical Findings.

Critical Findings
Literature provides limited, mixed evidence about associations between parental preconception adversity and offspring health in AA families
25 out of 30 unique studies reported significant associations between parental preconception adversity and offspring health; 6 out of 30 reported non-significant associations
Only six studies reported significant associations between parental preconception adversity and offspring health that was specific to AAs: 5 compared AAs who reported preconception adversity to AAs who did not, 1 compared AAs who reported preconception adversity to whites
Several studies reported both significant and nonsignificant associations across different offspring health outcomes

**Table 3.** Implications for Research, Practice, and Policy.

Implications
**Future research should:**
Account for paternal preconception adversity experiences when exploring intergenerational links to offspring health
Capture both general and race-specific parental preconception adversity (e.g., racism) disproportionately affecting African Americans using a diverse range of measures simultaneously
Measure offspring health beyond birth/early-life outcomes to examine longer-term repercussions of preconception adversity and identify mechanisms responsible for health repercussions
Conduct prospective, longitudinal studies that assess adversity and outcomes as they occur, not retrospectively
**Practitioners need to:**
Assess adversity to identify families at greatest risk for potential health impacts of adversity across generations in AA community
Conduct research to develop and test interventions that target the mechanisms linking parental preconception adversity with offspring health in the AA community
**Policy**
To address health disparities that affect AAs, funding is needed for rigorous longitudinal research examining the impact of parental preconception adversity on offspring health across the lifespan

Several potential mechanisms linking maternal adversity with offspring health were also suggested. In samples with only AAs, maternal preconception adversity was linked to both prenatal cortisol levels as well as changes in prenatal DBP that were ultimately associated with birth timing and birth weight (Gillespie et al., 2017; Hilmert et al., 2014). Studies including AAs, but not reporting findings exclusive to this group, identified multiple biomarkers (e.g., IL-6, placental tissue TL) as key mechanisms in the impact of maternal preconception adversity on several infant outcomes (e.g., admission to special care nursery, birth weight, length of hospital stay, PTB, small for gestational age). Lastly, some studies demonstrated significant links between maternal preconception adversity and prenatal behavioral and lifestyle mechanisms (e.g., smoking, substance use) that have been shown in previous work to partially explain negative outcomes for infants.

### Limitations of the Literature

The literature reviewed herein has several weaknesses that limit our ability to clearly address whether there is an association between parental preconception adversity and offspring health in AAs comprehensively (see Table 3). First, 25 (83%) of the 30 unique studies reviewed used relatively small convenience samples, introducing sampling and selection bias which renders the findings ungeneralizable to the broader AA population and limits the interpretation of any significant associations identified. These biases are further compounded by the fact that most studies did not adequately address important potential confounding variables (e.g., current parental mental/physical health status, child exposure to adversity) that may account for any significant associations identified.

Moreover, only six (20%) used an all-AA sample (comparing AAs with vs. without preconception adversity), and four of these studies had fewer than 100 participants (Gillespie et al., 2017; Hilmert et al., 2014; Jovanovic et al., 2011; Rowell, 2020); 60% of studies with multiracial samples had <50% AA respondents, highlighting concerns about sample size and potential sampling/selection bias. Studies using these relatively small, convenience samples also lack statistical power which, when combined with sampling/selection biases, limits the applicability of the findings. An additional five (17%) studies used multiracial samples and tested for racial differences, but most studies with multiracial samples (*n* = 19) did not test for racial differences. This is important because when interpreting both significant and non-significant findings from these studies, the degree to which these associations apply to AAs specifically and whether divergent findings for AAs are being obscured by larger racial groups within the samples is not clear. Finally, multiple articles reported data from the same nationally representative studies including the Asthma Coalition on Community Environment and Social Stress project (ACCESS; Brunst et al., 2017; Sternthal et al., 2011); the Early Childhood Longitudinal Study-Birth Cohort (Cheng et al., 2016; Witt et al., 2014a, 2014b, 2015, 2016); and the National Longitudinal Study of Adolescent to Adult Health (Add Health; Cammack et al., 2019; Flagg et al., 2014; Ihongbe, 2018; Strutz et al., 2014). Although the use of these nationally representative samples makes the reported article findings more generalizable, they each represent just one study with evidence linking different forms of parental adversity with different offspring outcomes because they come from the same sample.

In addition, the overwhelming majority of the 30 independent studies focused on birth and early-life outcomes (*n* = 26, 87%) providing limited evidence for the longer-term repercussions of parental adversity on offspring physical health. Although adverse birth outcomes may initiate a lifetime of poor health (Crume et al., 2014; Parkinson et al., 2013), it is not clear from these studies what role parental adversity plays in this process or what mechanisms might explain subsequent poor health. Knowing more about the root causes of offspring health outcomes and the mechanisms linking them with parental preconception adversity in AA families could inform the development of public health interventions seeking to interrupt the negative health effects of intergenerational transmission of trauma.

All but one of the studies included in this review (Noll et al., 2007) relied on retrospective parental reports of preconception adversity, which introduces substantial retrospective recall bias. Reports of distressing events from one’s past are subject to recall bias because respondents may not remember previous events accurately, may omit details or entire events, or unknowingly revise past memories, especially when the events being asked about happened several years before (Widom, 2019). Inaccurate reporting of past life events may prevent researchers from correctly identifying the specific parental adverse experiences associated with offspring health. Furthermore, social-desirability bias may result in underreporting these events despite being assured that their responses are anonymous or confidential due to a desire for their responses to be viewed favorably by others. Such underreporting may compromise the ability to detect potential associations with offspring outcomes. Assessments of parental preconception adversity were also quite disparate and this lack of consistency in measurement further limits our ability to draw conclusions about the types of parental adversity that may be more detrimental to child health. Finally, this literature currently suffers from sex-based, gender-role biases regarding the health impact of parental adversity as all included studies exclusively measured maternal (not paternal) adversity. This is a significant omission because recent work suggests that paternal preconception adversity can impact offspring health through genetic and epigenetic changes to sperm (Braun et al., 2017).

It is also important to note that 90% of studies in this review assessed universal forms of adversity (e.g., ACEs, overall lifetime adversity, neighborhood disadvantage) commonly experienced across all racial groups, while only three addressed race-based adversity. That is, very few studies addressed the link between parental preconception exposure to race-specific adversity (e.g., discrimination and racism) and offspring health. Past work documents that AAs experience these specific adversities in several life domains at disproportionate rates (DeGue et al., 2016; Williams & Collins, 2001) and they can be particularly damaging due to their complex nature. These adversities can occur on multiple levels (e.g., cultural, institutional, interpersonal), ultimately undermine positive views of the self, diminish social relationships and the sense of belonging, and interfere with overall quality of life (Brondolo et al., 2017). Furthermore, they include acute events that can also become persistent stressors when recurring instances occur over prolonged periods or when they produce additional adversity exposures and there are limited resources available to address them. Importantly, empirical work has suggested that the health impacts of these specific experiences may be transmitted across generations (Hill et al., 2017; Lewis et al., 2015). Given that AAs have historically experienced race-based adversity unlike that of most groups in the U.S. (except Indigenous Americans; e.g., slavery, segregation), there remains a need to address the impact of the unique adversities experienced by AA parents (e.g., anti-Black racism) on their offspring’s health if we are to fully interrogate the roots of racial health disparities seen today.

### Future Directions

This body of literature is underdeveloped in several ways, making it challenging to draw any strong conclusions. Future studies that include larger, nationally, or regionally representative AA samples are needed to increase the generalizability of findings. Alternatively, nationally representative multiracial, multiethnic samples could be used if researchers examine racial and ethnic differences in the associations between parental preconception adversity and offspring health. Beyond the use of retrospective methods, identifying populations as early in life as possible before conception takes place and following them longitudinally would be a more accurate way to measure adverse experiences and their impact on offspring health. In addition, more consistent efforts should be made to intentionally capture a diverse range of parental adversity during several distinct time periods (e.g., childhood, adulthood, preconception, and prenatal) within the same study and emphasize statistical analyses that provide opportunities to disentangle the intergenerational health impacts of adversity experienced at specific time periods in the parent’s life. For example, being able to account for the presence of prenatal adversity when exploring associations between preconception adversity (experienced in childhood, adolescence, and/or adulthood) and offspring health can help more accurately characterize the impact of preconception adversity and highlight potential mediating factors. This body of literature may also benefit from studies that employ a diverse range of measures (e.g., surveys or interviews, biological data) and utilize them simultaneously to capture the impact of adversity more comprehensively.

When it comes to offspring health, future studies should examine a wider array of outcomes to better understand the impact of parental preconception adversity. The current literature overwhelmingly addresses birth and infancy outcomes (e.g., weight, development); while they are important indicators of early life health, a more comprehensive assessment of health outcomes as children progress into adolescence and adulthood is needed to identify the long-term repercussions of parental preconception adversity across generations. By including health data that encompass the child’s developmental trajectory, investigators can access a greater assortment of physical health measures (e.g., biological, observational, survey) gathered directly from offspring, that are more accurate than parental reports, and may reflect intergenerational adversity’s health impact across the lifespan. Furthermore, it may provide measures that are more proximal to physical health abnormalities that can ultimately serve as indicators for some of the ailments and chronic diseases that disproportionately affect AA adults today (Carnethon et al., 2017; Mehta et al., 2013).

Future research should also examine how paternal experiences of preconception adversity may affect offspring health and the unique mechanisms that are responsible for this transmission from fathers to children. While some evidence suggests that maternal preconception adversity may be associated with offspring health trajectories (e.g., Mahrer et al., 2020), paternal preconception adversity may also affect offspring health. Focusing on fathers provides the advantage of also accounting for the potential impact of parental experiences on offspring health beyond the direct biological repercussions of maternal experiences through the uterine environment (Braun et al., 2017). Identifying and measuring potential mechanisms responsible for intergenerational transmission of health impacts by capturing biological measures (e.g., epigenetic changes, inflammatory biomarkers, cortisol, telomere length), behavioral (e.g., parental substance use), and other factors simultaneously should also be a strong focus, as well as how these factors may interact with maternal mechanisms to affect future generations’ health. Such work is essential to beginning to understand the intergenerational health impacts of paternal preconception adversity for AAs. More specifically, it may help us better understand how race-based adversities experienced disproportionately by AA boys and men (e.g., police encounters, incarceration) may be associated with offspring health relative to other, more general adversity (e.g., poverty, violence). Indeed, it is crucial for future studies to tease apart the unique impacts of different types of preconception adversity on offspring health so that the specific impact of racialized trauma on the intergenerational transmission of health disparities in AA families can be identified. Finally, it would allow us to address the unique impact of paternal adversity relative to maternal adversity, and how they interact to shape offspring health.

## Conclusion

This review provides mixed evidence about the intergenerational impacts of parental preconception adversity on offspring physical health in AA families. Most studies investigated general adversity (e.g., ACEs, early-life disadvantage) and birth-related outcomes rather than race-specific adversity (e.g., racism) and chronic diseases known to disproportionately affect AAs. Several potential mechanisms responsible for these intergenerational health impacts were also identified and measured. Most studies used multiracial samples without addressing racial differences or reporting findings exclusive to the AA population. Given the historical and ongoing adversity (e.g., racism, systemic violence) and health disparities experienced by AAs, exploring how preconception adversity may affect health across generations is essential. Doing so may help explain the many health disparities observed among the AA population.

## Supplemental Material

sj-pdf-1-tva-10.1177_15248380221074320 – Supplemental Material for Parental Preconception Adversity and Offspring Health in African Americans: A Systematic Review of Intergenerational Studies Click here for additional data file. Supplemental Material, sj-pdf-1-tva-10.1177_15248380221074320 for Parental Preconception Adversity and Offspring Health in African Americans: A Systematic Review of Intergenerational Studies by Josiah A. Sweeting, Adebisi Akinyemi and E. Alison Holman in Trauma, Violence, & Abuse
