# Supplementary material for: Parental Preconception Adversity and Offspring Health in African Americans: A Systematic Review of Intergenerational Studies
Source: Trauma Violence Abuse. 2022 Mar 4;24(3):1677–92. doi: 10.1177/15248380221074320 (PMC10240647; doi:10.1177/15248380221074320)
Supplement: sj-pdf-1-tva-10.1177_15248380221074320 – Supplemental Material for Parental Preconception Adversity and Offspring Health in African Americans: A Systematic Review of Intergenerational Studies [file sj-pdf-1-tva-10.1177_15248380221074320.pdf]

Supplemental Materials

Supplemental Appendix A. Keyword Search Terms

Race-terms- "african american" OR "african americans" OR "African Americans"[Mesh] OR blacks[tiab] OR afro-american OR afro american OR afro OR "black people" OR "People of Color" OR negro* OR "African Continental Ancestry Group"[Mesh] OR colored* OR "Race Factors"[Mesh] OR race[ti] OR races[ti] OR racial[ti] OR racially[ti] OR "Ethnic Groups"[Majr] OR ethnic[ti] OR ethnical*[ti]

Intergenerational-related terms- generation OR generations OR generational OR intergenerational OR inter-generational OR transgenerational OR trans-generational OR multigenerational OR multi-generational OR intergenerationality OR transmit OR transmitting OR transmission OR "Parents"[Mesh:NoExp] OR "Fathers"[Mesh] OR "Mothers"[Mesh] OR "Single Parent"[Mesh] OR maternal OR paternal OR mother OR father OR parental[tw] OR "Grandparents"[Mesh] OR grandparent* OR grand-parent* OR grandmother* OR grandfather* OR grandchild* OR granddaughter* OR grandson* OR grand-mother* OR grand-father* OR grand-child* OR grand-daughter* OR grand-son* OR child OR children OR childhood OR infant OR infants OR "Child"[Mesh] OR "Infant"[Mesh] OR "Adolescent"[Mesh] OR offspring* OR neighborhood* OR neighbourhood* OR "family history" OR "family medical history" OR "family histories" OR "family medical histories" OR fetal OR fetus OR preconception OR pediatric OR paediatric OR newborn* OR "Infant, Newborn"[Mesh] OR unborn

Adversity-related terms- "Gene-Environment Interaction"[Majr] OR "social discrimination"[Majr] OR "Social Segregation"[Majr] OR "Socioeconomic Factors"[Majr] OR "Stress Disorders, Traumatic"[Majr] OR "Stress, Physiological"[Majr:NoExp] OR "Stress, Psychological"[Majr] OR "Violence"[Majr] OR "Warfare"[Majr] OR "Working Poor"[Majr] OR

"Wounds and Injuries"[Majr] OR abuse[ti] OR abused[ti] OR accident[ti] OR accidental[ti] OR accidents[ti] OR adverse[ti] OR adversity[ti] OR aggress[ti] OR aggression[ti] OR aggressive[ti] OR altercation[ti] OR assault[ti] OR attack[ti] OR attacks[ti] OR attacked[ti] OR attacking[ti] OR bereavement[ti] OR bully[ti] OR burden*[ti] OR childhood maltreatment[ti] OR coerce*[ti] OR coercive[ti] OR danger*[ti] OR death[ti] OR deprivation[ti] OR deprived[ti] OR destruct[ti] OR destructing[ti] OR destructive[ti] OR disadvantage*[ti] OR disaster[ti] OR discriminate[ti] OR discrimination[ti] OR discriminatory[ti] OR dislocation[ti] OR economic environment[ti] OR economic[ti] OR economically[ti] OR economics[ti] OR exploit[ti] OR exploitation[ti] OR exploited[ti] OR exposure[ti] OR exposures[ti] OR financial[ti] OR frighten[ti] OR gene-environment*[ti] OR genocidal[ti] OR genocide[ti] OR grief[ti] OR grieving[ti] OR hardship*[ti] OR harm[ti] OR harmed[ti] OR harmful[ti] OR harmfulness[ti] OR harming[ti] OR harms[ti] OR homeless[ti] OR homicide[ti] OR humiliate[ti] OR humiliated[ti] OR humiliation[ti] OR incarcerate[ti] OR incarceration[ti] OR intimidate[ti] OR intimidating[ti] OR lose[ti] OR loss[ti] OR losses[ti] OR lost[ti] OR maltreatment[ti] OR neglect[ti] OR neglected[ti] OR neglectful[ti] OR neglecting[ti] OR pain[ti] OR poor[ti] OR poverty[ti] OR prison[ti] OR punishment[ti] OR punishments[ti] OR racism[ti] OR rape[ti] OR relocation[ti] OR segregat*[ti] OR shooting[ti] OR shot[ti] OR slavery[ti] OR socio-economic status[ti] OR socioeconomic status[ti] OR stress[ti] OR stressed[ti] OR stressor[ti] OR terror[ti] OR terrorized[ti] OR terrors[ti] OR threat[ti] OR threatening[ti] OR threats[ti] OR trauma[ti] OR traumas[ti] OR traumatic[ti] OR traumatisation[ti] OR traumatised[ti] OR traumatization[ti] OR traumatized[ti] OR violence[ti] OR violent[ti] OR war[ti] OR worried[ti] OR worries[ti] OR worry[ti] OR wound[ti] OR wounded[ti] OR wounds[ti]

Physical health terms- "acute disease"[ti] OR "Acute Disease"[Majr] OR "acute diseases"[ti] OR "Allostasis"[Mesh] OR "Arthritis"[Majr] OR "birth outcome"[ti] OR "birth outcomes"[ti] OR "birth weight"[ti] OR "blood pressure"[ti] OR "Blood Pressure"[Mesh] OR "Bronchial Diseases"[Majr] OR "Cardiovascular Diseases"[Majr] OR "chronic disease"[ti] OR "Chronic Disease"[Majr] OR "chronic diseases"[ti] OR "Diabetes Mellitus"[Majr] OR "Fatigue"[Majr:NoExp] OR "Female Urogenital Diseases"[Majr] OR "Fetal Death"[Majr] OR "Fetal Mortality"[Majr] OR "Gastrointestinal Diseases"[Majr] OR "Headache"[Majr:NoExp] OR "health care" disparities[ti] OR "health care" disparity[ti] OR "health care" inequalities[ti] OR "health care" inequality[ti] OR "health disparities"[ti] OR "health disparity"[ti] OR "health outcome"[ti] OR "health outcomes"[ti] OR "health status"[ti] OR "heart failure"[ti] OR "heart murmur"[ti] OR "heart murmurs"[ti] OR "Hemophilia A"[Majr] OR "Hemophilia B"[Majr] OR "Hypersensitivity"[Majr] OR "Hypertension"[Majr] OR "Infant Mortality"[Majr] OR "Infection"[Majr] OR "Liver Cirrhosis"[Majr] OR "Male Urogenital Diseases"[Majr] OR "Migraine Disorders"[Majr] OR "minorities health"[ti] OR "minority health"[ti] OR "Minority Health"[Majr] OR "Morbidity"[Majr] OR "Mortality"[Majr] OR "multiple sclerosis"[ti] OR "Musculoskeletal Diseases"[Majr] OR "Neoplasms"[Majr] OR "Nervous System Diseases"[Majr] OR "Neuroanatomy"[Majr] OR "Neurochemistry"[Majr] OR "Neuropathology"[Majr] OR "Obesity"[Majr] OR "Outcome Assessment (Health Care)"[Majr:NoExp] OR "outcome assessment"[ti] OR "Pain"[Majr] OR "peptic ulcer"[ti] OR "peptic ulcers"[ti] OR "Perinatal Death"[Majr] OR "Peripartum Period"[Majr] OR "physical ailment"[ti] OR "physical ailments"[ti] OR "physical health"[ti] OR "physical outcome"[ti] OR "physical outcomes"[ti] OR "Postpartum Period"[Majr] OR "Pregnancy"[Majr] OR "Premature Birth"[Majr] OR "Pulmonary Emphysema"[Majr] OR "Respiration Disorders"[Majr] OR

"Risk"[Majr] OR "Seizures"[Majr] OR "Signs and Symptoms, Respiratory"[Majr] OR "Social Determinants of health"[Majr] OR "Stroke"[Majr] OR allergic[ti] OR allergies[ti] OR allergy[ti] OR allostatic[ti] OR ante-natal*[ti] OR ante-partum[ti] OR antenatal*[ti] OR antepartum[ti] OR arrhythmia[ti] OR arthritis[ti] OR asthma*[ti] OR birth-weight*[ti] OR birthweight*[ti] OR blood[ti] OR bowel[ti] OR bronchitis[ti] OR cancer[ti] OR cardiac[ti] OR cardio[ti] OR cardiovascular[ti] OR circulatory[ti] OR cirrhosis[ti] OR colitis[ti] OR diabetes[ti] OR emphysema[ti] OR endocrine[ti] OR epigenetic*[ti] OR epigenomic[ti] OR epigenomics[ti] OR fatigue*[ti] OR gastrointestinal[ti] OR genitourin*[ti] OR headache*[ti] OR healthcare disparities[ti] OR healthcare disparity[ti] OR healthcare inequalities[ti] OR healthcare inequality[ti] OR hemophilia[ti] OR hypertension[ti] OR immune[ti] OR incongruit* OR infection[ti] OR infections[ti] OR infectious[ti] OR inflammatory[ti] OR intrauterine[ti] OR migraine*[ti] OR morbidities[ti] OR morbidity[ti] OR mortalities[ti] OR mortality[ti] OR musculoskeletal[ti] OR myocardial[ti] OR neonatal[ti] OR neuroanatomy[ti] OR neurologic*[ti] OR neurological[ti] OR neuropathology[ti] OR obesity[ti] OR pain[ti] OR palpitation*[ti] OR perinatal*[ti] OR peri-natal*[ti] OR pregnan*[ti] OR premature[ti] OR pre-mature[ti] OR prenatal[ti] OR pre-natal[ti] OR preterm[ti] OR pre-term OR reproduc*[ti] OR reproduction[ti] OR reproductive[ti] OR respiration[ti] OR respiratory[ti] OR risks[ti] OR risky[ti] OR seizure*[ti] OR still-birth*[ti] OR still-born*[ti] OR stillbirth*[ti] OR stillborn*[ti] OR stroke[ti] OR strokes[ti] OR tumor*[ti] OR tumour*[ti] OR ulcer[ti] OR ulcers[ti] OR weathering OR well-being[ti] OR well-ness[ti] OR wellbeing[ti] OR wellness[ti] OR wheez*[ti] OR "metabolic syndrome"[ti] OR "Metabolic Syndrome"[Majr] OR "high cholesterol"[ti] OR "Hypercholesterolemia"[Majr] OR hyperlipidemia[ti] OR "Hyperlipidemias"[Majr] OR "irritable bowel syndrome"[ti] OR "Irritable Bowel Syndrome"[Majr] OR "inflammatory bowel

disease"[ti] OR "Inflammatory Bowel Diseases"[Mesh] OR "crohn's disease"[ti] OR

autoimmune[ti] OR "Autoimmune Diseases"[Majr]

Supplemental Appendix B. Articles with parental adversity measured clearly *before* pregnancy (full AA sample)

Study & Sample	Key Measures	Key Results	Risk of Bias (ROB)
Study: Gillespie et al., 2017<br><br>Sample: 96 pregnant African-American women and their infants<br><br>Design: Prospective cohort	Predictor: Cumulative maternal childhood stress measured using STRAIN<br><br>Outcomes: Infant birth timing; Infant birth following spontaneous labor<br><br>Mediator: Prenatal maternal plasma cortisol	- ↑ Maternal cumulative childhood stress → earlier birth timing (controls: adult stress, cortisol)<br><br>- Maternal cortisol mediated link between childhood stress and earlier birth timing in women who had spontaneous labor<br><br>Mechanism of Transmission: Childhood stress alters birth outcomes through prenatal maternal cortisol	ROB: Moderate; primary source of bias was non-representative sample; maternal preconception adversity assessed retrospectively
Study: Hilmert et al., 2014<br><br>Sample: 39 pregnant African-American women and their infants<br><br>Design: Retrospective longitudinal cohort	Predictor: Maternal lifetime racism<br><br>Outcomes: Infant BW and GA via medical charts<br><br>Moderators: Maternal prenatal SBP and DBP	- 2+ domains of maternal exposure to indirect racism in childhood → ↓BW as mom's prenatal DBP↑ (controls: BMI, SES, and SLEI)<br><br>Mechanisms of Transmission: Maternal racism exposure affects birth outcomes through prenatal BP	ROB: Moderate; primary source of bias was non-representative sample used; maternal preconception adversity assessed retrospectively
Study: Jovanovic et al., 2011<br><br>Sample: 36 African-American children 6-13 years old and their mothers<br><br>Design: Retrospective cross-sectional	Predictors: Maternal exposure to perceived childhood emotional, physical, and sexual abuse using CTQ<br><br>Outcomes: Child startle response; Child HRV acquired via electrocardiogram	- Maternal physical abuse → ↑child dark-enhanced startle (controls: child age, sex)<br><br>- ↑ vs. ↓Maternal emotional abuse → ↑child LF/HF ratios	ROB: High; primary sources of bias were small, non-representative sample and participants' response rate not reported; maternal preconception adversity assessed retrospectively

Study: Rowell, 2020<br><br>Sample: 31 expectant African-American mothers<br><br>Design: Retrospective cross-sectional	Predictor: Maternal ACEs using ACEs Questionnaire<br><br>Outcomes: Infant BW and GA as reported by doulas at birth	- Maternal ACEs not significantly associated with GA or BW (controls: maternal age)	ROB: High; primary sources of bias were small, non-representative sample and participants' response rate not reported; maternal preconception adversity assessed retrospectively
Study: Sealy-Jefferson et al., 2019<br><br>Sample: 1365 African-American women and their infants<br><br>Design: Retrospective cohort	Predictor: Current PS during past month using PSS<br><br>Outcome: Infant PTB<br><br>Moderators: Early-life neighborhood social disorder; Early-life neighborhood social control	- ↑Early-life neighborhood social disorder and ↑current stress → ↑odds of PTB relative to ↓early-life social disorder (controls: maternal age, marital status, education, income)<br><br>- ↓early-life neighborhood social disorder → No association between PS and PTB	ROB: Moderate; primary source of bias was non-representative sample used; maternal preconception adversity assessed retrospectively

Supplemental Appendix C. Articles with parental adversity measured clearly *before* pregnancy (partial AA sample testing for racial differences)

Study & Sample	Key Measures	Key Results	Risk of Bias (ROB)
Study: Dominguez et al., 2008<br><br>Sample: 124 pregnant women (41.1% African American) and their infants<br><br>Design: Prospective longitudinal cohort	Predictor: Race using self-identification as "African American or Black" or "Non-Hispanic White"<br><br>Outcomes: Infant BW and GA using medical charts<br><br>Mediators: Maternal childhood and adulthood direct/vicarious exposure to racism	- Mom's lifetime and vicarious childhood exposure to racism →↓BW for Black moms only (controls: parents' childhood education)<br><br>- No group differences in GA	ROB: Moderate; primary source of bias was the non-representative sample; maternal preconception adversity assessed retrospectively
Study: Gray et al., 2017<br><br>Sample: 167 infants (49% female) and their mothers (61% African American)<br><br>Design: Retrospective cohort	Predictor: Maternal exposure to ACEs using ACEs survey<br><br>Outcomes: 4-mo old infant RSA measured during dyadic play and dyadic completion of the SFP<br><br>Moderators: Infant sex and race using maternal report	- ↑Maternal ACEs → ↓infant RSA during dyadic play (controls: infant sex, race)<br><br>- ↑Maternal ACEs → ↓infant RSA during SFP<br><br>- No significant sex or race differences found in infant RSA	ROB: High; primary sources of bias were lack of information about participants' follow-up rate and the non-representative sample; maternal preconception adversity assessed retrospectively
Study: Margerison-Zilko et al., 2017<br><br>Sample: 2,559 women (21% African American) and their infants<br><br>Design: Retrospective cohort	Predictors: Maternal childhood, adulthood SLE using Turner, Wheaton, and Lloyd Checklist; events scored as never, in childhood, in adulthood, or both<br><br>Outcomes: Infant early and late PTB<br><br>Moderator: Maternal race/ethnicity	- Maternal abuse/violence in childhood only vs. never → ↑late PTB (controls: race/ethnicity, education, parity, marital status)<br><br>- Race/ethnicity did not moderate the association between SLE and PTB or PTB by timing	ROB: Moderate; primary source of bias was the non-representative sample; maternal preconception adversity assessed retrospectively
Study: Masho et al., 2015<br><br>Sample: 231 pregnant women (72% African American) and their infants	Predictors: Maternal lifetime and past year SLE using SLEI; Maternal PS in her life, past year, and last month using PSS; Maternal prenatal cortisol via saliva samples	- Lifetime exposure to SLE was not associated with PTB in either the AA subsample or the full sample (controls: maternal age, education, adequacy of prenatal care)	ROB: High; primary sources of bias were participants' follow-up rate not reported and non-representative sample; maternal preconception adversity

<u>Design</u>: Retrospective cohort	<u>Outcome</u>: Infant PTB		assessed retrospectively
<u>Study</u>: Seng et al., 2011<br><br><u>Sample</u>: 839 women (41.4% African American) and their infants<br><br><u>Design</u>: Prospective longitudinal cohort	<u>Predictor</u>: Current and lifetime maternal PTSD diagnoses using National Women's Study PTSD Module<br><br><u>Outcomes</u>: Infant BW and GA<br><br><u>Moderator</u>: Maternal childhood abuse using LSC	- Maternal childhood abuse not related to infant BW or GA (controls: comorbidity, risk behaviors, medical and obstetric risk factors, modifiable health care related factors, chronic stress)<br><br>- Among women who experienced child abuse, African-American race was the strongest predictor of LBW	<u>ROB</u>: High; primary sources of bias were the non-representative sample and inadequate % of participants retained at follow up; maternal preconception adversity assessed retrospectively

Supplemental Appendix D. Articles with parental adversity measured clearly *before* pregnancy (partial AA sample not testing racial differences)

Study & Sample	Key Measures	Key Results	Risk of Bias (ROB)
Study: Blackmore et al., 2016<br><br>Sample: 358 pregnant women (49.7% African American) and their infants<br><br>Design: Prospective cohort	Predictors: Symptoms of maternal depression and anxiety using EPDS and PSWQ, respectively<br><br>Outcomes: Infant BW and GA<br><br>Moderator: Traumatic events exposure using PTSD section of the SCID	- ↑Anxiety among women who experienced childhood trauma → ↓BW (controls: maternal ethnicity, BMI, prenatal alcohol use and smoking, pregnancy history, SES)<br><br>- Maternal trauma, depression, and anxiety were not linked to GA	ROB: Moderate; primary source of bias was the non-representative sample; maternal preconception adversity assessed retrospectively
Study: Chen et al., 2017<br><br>Sample: 150 children aged 9 to 17 years (25% African American) with physician diagnosed asthma and a parent<br><br>Design: Retrospective cross-sectional	Predictor: Parents' childhood SES using early childhood home ownership (renting=↓SES; owning=↑SES)<br><br>Outcomes: ACT completed by children and parents; child T_H2 and T_H1 cytokine production	- Lower parental childhood SES → ↓child asthma control vs. higher parental childhood SES (controls: child age, sex, ethnicity, usage of beta agonists and inhaled corticosteroids)<br><br>- ↓Parental childhood SES → ↑child T_H2 and T_H1 cytokine production vs. offspring of ↑parental SES	ROB: Moderate; primary source of bias was the non-representative sample; maternal preconception adversity assessed retrospectively
Study: Cheng et al., 2016<br><br>Sample: 6,900 children and their mothers (14.3% African American)<br><br>Design: Retrospective cohort	Predictor: Maternal PSLEs<br><br>Outcomes: VLBW infant; Maternal reported infant/toddler health at 9 and 24 months including overall health status, clinically diagnosed SHCN, and any severe health condition	- ↑Maternal PSLEs → ↑odds VLBW infant (controls: maternal chronic conditions, # of children, parity, age, race/ethnicity, marital status, insurance status, SES, region, pregnancy complications, pre-pregnancy BMI, initiation of prenatal care)<br><br>- ↑Maternal PSLEs → poorer child health status, ↑odds SHCN, and ↑severe health conditions at 9 mo	ROB: Low; primary source of bias was maternal preconception adversity assessed retrospectively
Study: Cowell et al., 2021<br><br>Sample: 829 mother-	Predictors: Maternal childhood IPT using CTQ; Maternal lifetime trauma and non-traumatic stress in prior year	- Maternal childhood IPT not associated with PTB risk (controls: maternal ethnicity, age, parity,	ROB: Moderate; primary source of bias was the non-representative sample;

newborn pairs (45% African American)<br><br>Design: Prospective cohort	using LSC-R; Maternal trait anger expression using STAXI-2 subscales<br><br>Outcome: Infant GA	relationship status, education level, prenatal smoking, pre-pregnancy BMI)	maternal preconception adversity assessed retrospectively
Study: Freedman et al., 2017<br><br>Sample: 133 women experiencing stillbirth (17% African American) and 500 women delivering a healthy term live birth (12% African American)<br><br>Design: Case-control	Predictors: Maternal childhood maltreatment using CTQ with physical abuse, CSA, emotional abuse, physical neglect, and emotional neglect subscales<br><br>Outcome: Infant stillbirth status gathered from medical records	- Maternal childhood emotional neglect → ↑infant stillbirth risk (controls: maternal age, education)<br><br>- No other forms of maternal maltreatment significantly associated with stillbirth risk	ROB: Moderate; primary source of bias was the different participant response rates in the case and control groups; maternal preconception adversity assessed retrospectively
Study: Jones et al., 2019<br><br>Sample: 67 pregnant mothers (56.7% African American) and their four-month-old infants (43.3% female)<br><br>Design: Prospective cohort	Predictor: Maternal ACEs using ACEs Questionnaire<br><br>Outcome: Infant RSA stress responsivity<br><br>Moderator: Placental TL	- ↑Maternal ACEs → ↑infant RSA stress responsivity (controls: sex, race, maternal prenatal smoking)<br><br>- ↑Maternal ACEs → shorter placental TL<br><br>- Placental TL and maternal ACEs interacted to predict both infant RSA reactivity and recovery<br><br>Mechanism of Transmission: Maternal ACEs → changes in placental TL → ↑infant stress responsivity	ROB: High; primary sources of bias were the lack of information about participants' follow-up rate and non-representative sample; maternal preconception adversity assessed retrospectively
Study: Mersky et al., 2019<br><br>Sample: 1848 women (24% African American) with children<br><br>Design: Retrospective cohort	Predictor: Maternal ACEs using CES with responses summed & categorized (0, 1-2, 3-4, and 5+ ACEs)<br><br>Outcomes: Pregnancy loss (e.g., miscarriage or still birth), PTB, and LBW using archival program records	- ↑Maternal ACEs → ↑odds of pregnancy loss, PTB, and LBW (controls: maternal age, race/ ethnicity, educational attainment)<br><br>- 5+ ACEs → ↑odds of pregnancy loss; no differences in PTB or LBW	ROB: High; primary sources of bias were the lack of information about participants' response rate and non-representative sample; maternal preconception adversity assessed

		odds<br><br>- No differences in birth outcomes between mothers with 0 ACEs and those with 1-2 or 3-4 ACEs	retrospectively
Study: Miller et al., 2017<br><br>Sample: 744 pregnant women (16.3% African American) and their infants<br><br>Design: Prospective cohort	Predictor: Maternal childhood economic hardship<br><br>Outcomes: Infant birth outcomes using medical charts including length of gestation (e.g., PTB), fetal growth (e.g., BW; SGA), length of hospital stay, and SCN<br><br>Mediators: Maternal inflammatory biomarkers (e.g., IL-6, IL-8); Psychosocial pathways (e.g., maternal education); Lifestyle pathways (e.g., pre-pregnancy BMI); Obstetric pathways (e.g., history of PTB)	- ↑Maternal childhood disadvantage → ↑odds of adverse birth outcomes (controls: age, race/ethnicity, nulliparity, gestational hypertension, pre-eclampsia, PTB history)<br><br>- Maternal childhood disadvantage → ↑pre-pregnancy BMI → ↑Maternal IL-6 levels → adverse birth outcomes<br><br>- Maternal childhood disadvantage → ↓Maternal education & ↑pre-pregnancy BMI → adverse birth outcomes<br><br>Mechanisms of Transmission: Maternal disadvantage → maternal inflammatory, psychosocial, lifestyle, and obstetric factors → adverse birth outcomes	ROB: Moderate; primary source of bias was non-representative sample; maternal preconception adversity assessed retrospectively
Study: Noll et al., 2007<br><br>Sample: 67 offspring (~49% African American) born to mothers who experienced CSA and 56 offspring (46% African American) born to nonabused comparison mothers	Predictor: Maternal CSA determined by records of substantiated contact CSA from CPS agencies<br><br>Outcome: Infant PTB using hospital records<br><br>Mediators: Maternal salivary cortisol; Maternal prenatal alcohol use reported in labor and delivery records	- Maternal CSA → ↑odds of PTB status (controls: minority status, sibling number)<br><br>- Maternal prenatal alcohol use partially mediated link between maternal CSA and PTB<br><br>Mechanisms of Transmission: Maternal prenatal alcohol use	ROB: Moderate; primary source of bias was non-representative sample

Design: Prospective cohort		partially mediated link between maternal CSA and PTB status	
Study: Smith et al., 2016<br><br>Sample: 2303 pregnant women (7% African American) and their infants<br><br>Design: Prospective cohort	Predictor: Maternal ACEs using ETI-SF<br><br>Outcomes: PTB and LBW<br><br>Mediators: Maternal prenatal smoking and substance use via interview	- ↑Maternal ACEs →↑LBW and ↓GA (controls: maternal marital status, prenatal illicit substance use and alcohol use, SRI use, psychiatric disorder, education, smoking, social support)<br><br>Mechanisms of Transmission: Maternal prenatal smoking and substance use mediated impact of ACEs on BW; Prenatal smoking was the strongest mediator of the impact of ACEs on GA	ROB: Moderate; primary source of bias was non-representative sample; maternal preconception adversity assessed retrospectively
Study: Sternthal et al., 2011<br><br>Sample: 510 pregnant women (28% African American) and their infants<br><br>Design: Prospective cohort	Predictor: Maternal childhood SES using parental home ownership from birth to age 10<br><br>Outcomes: Child cord blood IgE levels (IU/mL) using CAP fluorescent enzyme immunoassay; Maternal report of infant wheezing at 2 years old<br><br>Mediators: Social pathways (e.g., maternal IPT exposure); Physical pathways (e.g., prenatal household allergens)	- ↓Maternal childhood SES → ↑cord blood IgE levels (controls: child sex, maternal race/ethnicity, atopy, nativity status)<br><br>- No mediators linking maternal childhood SES and cord blood IgE<br><br>- Maternal lifetime IPT → ↑cord blood IgE; maternal childhood SES not related to maternal IPT<br><br>- Significant indirect effects linking low maternal childhood SES and child wheeze via adult SES and prenatal environmental exposures	ROB: Moderate; primary source of bias was non-representative sample; maternal preconception adversity assessed retrospectively
Study: Witt et al., 2014a<br><br>Sample: 9,350 children and their mothers (14.1% African American)	Predictor: Maternal PSLEs<br><br>Outcomes: Infant LBW and VLBW	- Any maternal PSLEs vs. no PSLEs → ↑odds VLBW infant (controls: see Cheng et al. 2016)<br>- Maternal PSLEs not linked w/LBW	ROB: Low; primary source of bias was retrospective measure of maternal preconception adversity

Design: Retrospective cohort		- Timing of PSLEs exposure affected associations such that PSLEs ≥ 1 year pre-conception → ↑odds of VLBW baby	
Study: Witt et al., 2014b<br><br>Sample: 9,350 children and their mothers (14.1% African American)<br><br>Design: Retrospective cohort	Predictor: Maternal PSLEs<br><br>Outcome: Infant PTB<br><br>Moderator: Maternal age	- Maternal PSLEs and age interacted to predict PTB: younger women with PSLE → ↑ PTB risk vs. older women (controls: see Cheng et al. 2016)<br><br>- Women aged 20-24 or 30 years or older exposed to PSLEs 1 year or more prior to conception had ↑ PTB risk than women aged 25-29 years without such an event	ROB: Low; primary source of bias was retrospective measure of maternal preconception adversity
Study: Witt et al., 2015<br><br>Sample: 9,300 children and their mothers (14% African American)<br><br>Design: Retrospective cohort	Predictor: Maternal PSLEs<br><br>Outcome: Infant LBW and VLBW<br><br>Moderator: Maternal neighborhood disadvantage	-↑Maternal PSLEs → ↑risk of VLBW (controls: see Cheng et al. 2016)	ROB: Low; primary source of bias was retrospective measure of maternal preconception adversity
Study: Witt et al., 2016<br><br>Sample: 9,350 children and their mothers (14.1% African American)<br><br>Design: Retrospective cohort	Predictor: Maternal PSLEs<br><br>Outcome: Infant LBW and VLBW	- ↑Maternal PSLEs →↑risk VLBW vs. no PSLEs (controls: see Cheng et al. 2016)<br>- PSLE exposure → ↑ risk LBW	ROB: Low; primary source of bias was retrospective measure of maternal preconception adversity

Supplemental Appendix E. Articles with parent-reported offspring health outcomes

Study & Sample	Key Measures	Key Results	Risk of Bias (ROB)
Study: Astone et al., 2007<br><br>Sample: 987 infant (G3), mother (G2), and grandmother (G1) groups (82.5% African American)<br><br>Design: Prospective cohort	Predictors: Grandmother's (G1) education; Maternal (G2) childhood household income; G2 family structure; G2's household receipt of public assistance at birth or age 7<br><br>Outcome: Maternal reported infant BW (G3)	- If mother was poor → ↑risk LBW (controls: G3 sex, G2 adult height, multipara, prenatal smoking, difference between G2 BW & G1 BW, G1 pre-pregnancy BMI, infant BWs, STDs, prenatal smoking)<br>- ↑ income/needs ratio → ↑ BW	ROB: Moderate; primary source of bias was non-representative sample; maternal report of offspring health
Study: Brunst et al., 2017<br><br>Sample: 857 pregnant women (30% African American) and their infants<br><br>Design: Prospective cohort	Predictors: Maternal lifetime IPT using R- CTS: unexposed, child/adolescent IPT, adult/index pregnancy IPT, or chronic IPT<br><br>Outcome: Maternal report of MD-diagnosed asthma from birth up to age six years<br><br>Mediator: Maternal prenatal asthma	- Chronic maternal IPT vs. no IPT → ↑male child asthma risk, (controls: maternal age, education, child sex & birthweight, race/ethnicity)<br><br>- Early life IPT not linked with child asthma<br><br>- Maternal prenatal asthma mediated link between chronic IPT and child asthma	ROB: Moderate; primary source of bias was non-representative sample; retrospective measure of maternal preconception adversity and maternal report of offspring health
Study: Cammack et al., 2019<br><br>Sample: 4,181 female adolescents (18.2% African American) and their infants<br><br>Design: Retrospective cohort	Predictors: Maternal childhood abuse and age each abuse first occurred<br><br>Outcomes: Maternal reported infant PTB and VPTB	- Maternal CSA exposure between ages 9-18 by non-parental/adult caregivers using physical force → ↑VPTB risk (controls: race, childhood SES)	ROB: Low; primary source of bias was retrospective measure of maternal preconception adversity; maternal report of offspring health
Study: Daniels et al., 2020<br><br>Sample: 208 African-American women and their infants<br><br>Design: Retrospective, cross-sectional	Predictors: Direct and vicarious racial discrimination in childhood, adolescence, & adulthood<br><br>Outcome: Maternal reported infant PTB	- ↑Adolescent direct racial discrimination → ↑PTB risk (controls: # of pregnancies, education, employment status, marital status)<br><br>- ↑Childhood vicarious racial discrimination → ↑PTB risk	ROB: High; primary sources of bias were non-representative sample and inadequate participants' response rate information; retrospective measure of maternal preconception adversity and maternal report

			of offspring health
Study: Flagg et al., 2014<br><br>Sample: 535 adolescent mothers (28% African American), their parents, and their infants<br><br>Design: Retrospective cohort	Predictor: Grandparental perceived neighborhood disorder<br><br>Outcome: Maternal reported infant BW	- Grandparental exposure to neighborhood disorder not linked to grandchild's BW (controls: grandparent education, maternal race, age, BW, prenatal care, drug use, PTB)	ROB: Low; primary source of bias was retrospective measure of maternal preconception adversity; maternal report of offspring health
Study: Freeman, 2014<br><br>Sample: 2,332 mothers (43.7% African American) and their infants<br><br>Design: Retrospective cohort	Predictor: Grandmother report of maternal early life poverty<br><br>Outcome: Maternal reported infant birthweight, with LBW	- Maternal early life poverty not associated with LBW (controls: maternal race, infant sex, maternal health	ROB: Moderate; primary source of bias was inadequate participants' response rate information; maternal report of offspring health
Study: Gavin et al., 2011<br><br>Sample: 136 mother-child dyads (26% African American)<br><br>Design: Retrospective cohort	Predictors: Maternal childhood maltreatment using CTQ; Maternal childhood low SES<br><br>Outcome: Maternal reported BW<br><br>Mediators: Maternal adolescent substance use; Maternal prenatal tobacco and alcohol use	- Maternal low childhood SES → ↓BW (controls: maternal substance use)<br><br>Mechanisms of Transmission:<br>- Maternal early childhood maltreatment → ↑adolescent substance use and ↑prenatal tobacco and alcohol use → ↑risk LBW	ROB: Moderate; primary source of bias was non-representative sample; retrospective measure of maternal preconception adversity and maternal report of offspring health
Study: Hillis et al., 2004<br><br>Sample: 9,159 women (4.7% African American) and their infants<br><br>Design: Retrospective cohort	Predictor: Maternal ACEs<br><br>Outcome: Maternal reported pregnancy outcome (e.g., live birth, stillbirth/miscarriage)	- ↑Maternal ACEs → ↑risk of fetal death in 1st pregnancy (controls: maternal age, race, education, adolescent pregnancy)<br><br>- In 2nd pregnancy, ↑ maternal ACEs → ↑ risk of fetal death<br><br>- If 1st pregnancy as teen → no elevated risk of fetal death	ROB: High; primary sources of bias were non-representative sample used and inadequate participant response rate obtained; retrospective measure of maternal preconception adversity and maternal report of offspring health

Study: Ihongbe, T. O.<br><br>Sample: 4,419 women (20.7% African American) and their infants<br><br>Design: Retrospective cohort	Predictor: Maternal exposure to neighborhood violence in study waves prior to the delivery of their infant<br><br>Outcome: Maternal reported PTB<br><br>Moderator: Maternal social support	- ↑maternal exposure to neighborhood violence → ↑PTB risk vs. women not exposed to neighborhood violence (controls: maternal age, insurance status, marital status, household income, prenatal alcohol use)<br><br>- Social support did not moderate association	ROB: Low; primary source of bias was retrospective measure of maternal preconception adversity; maternal report of offspring health
Study: Kerkar et al., 2020<br><br>Sample: 1,511 women (63.3% African American) and their infants<br><br>Design: Retrospective cohort	Predictors: Maternal ACEs using ACEs survey<br><br>Outcomes: Maternal reported pregnancy outcome (e.g., MAP; MFP)	- ↑Maternal ACEs → ↑risk of MFP and MAP (controls: maternal age at pregnancy, race, BMI, education, marital status, smoking)	ROB: High; primary sources of bias were inadequate participants' response rate information and non-representative sample; retrospective measure of maternal preconception adversity and maternal report of offspring health
Study: Lê-Scherban et al., 2018<br><br>Sample: 350 parent-child dyads (45.1% African American; 80% adult women)<br><br>Design: Retrospective cross-sectional	Predictors: Parental exposure to ACEs using ACE study and Behavioral Risk Factor Surveillance Survey ACE module; Parental community-based childhood stress<br><br>Outcomes: Proxy report (92% parent) of child health outcomes	- ↑Parental ACEs → ↑risk of poor offspring health not related to risk of obesity or asthma (controls: parent age, sex, race/ethnicity, child age, sex)<br><br>- ↑Parental expanded ACE exposure → ↑odds of poorer offspring health, obesity, and asthma	ROB: Moderate; primary source of bias was inadequate participant response rate obtained; retrospective measure of maternal preconception adversity and maternal report of offspring health
Study: Stein et al., 2000<br><br>Sample: 974 homeless women (57.4% African American) and their infants<br><br>Design: Retrospective,	Predictors: Maternal history of rape or CSA before age 18; Maternal assault before age 18<br><br>Outcomes: Maternal reported PTB & LBW	- Women reporting rape or CSA before age 18 → ↑ PTB risk and ↓GA vs. no rape/CSA; (controls: ethnicity, income)<br><br>- No significant difference in PTB and LBW risk for women reporting	ROB: Low; primary source of bias was retrospective measure of maternal preconception adversity; maternal report of offspring health

cross-sectional		an assault before age 18	
Study: Strutz et al., 2014<br><br>Sample: 3,512 1st-time (23.7% African American) and 1,901 (25.5% African American) 2nd-time mothers<br><br>Design: Retrospective cohort	Predictors: Maternal PSLEs in adolescence and emerging adulthood; Maternal PSLEs pertaining to family of origin and early experiences<br><br>Outcome: Maternal report infant BW	- ↑ Chronic maternal PSLEs → ↑ risk LBW in 1st & 2nd births (controls: age, parity, BMI, smoking, alcohol, marital status)<br><br>- Acute maternal PSLEs not linked with infant BW	ROB: Low; primary source of bias was retrospective measure of maternal preconception adversity; maternal report of offspring health

Abbreviations for table: ACEs=Adverse Childhood Experiences; ACT=Asthma Control Test; BMI=body mass index; BW=Birth weight; CES=Childhood Experiences Survey; CTQ=Childhood Trauma Questionnaire; DBP= diastolic blood pressure; EPDS=Edinburgh Postnatal Depression Scale; ETI-SF=Early Trauma Inventory Self Report Short Form; GA=gestational age; HRV=heart-rate variability; IPT= interpersonal trauma; LBW=low birth weight (< 2500 grams or 5.5 pounds); LSC-R=Life Stressor Checklist-Revised; MAP=miscarriage at any pregnancy; MFP=miscarriage at first pregnancy; NDI=neighborhood disadvantage index; PS=perceived stress; PSLEs= stressful life events prior to conception; PSS=Cohen's Perceived Stress Scale; PSWQ=Penn State Worry Questionnaire; PTB=preterm birth (birth < 37 completed weeks gestation); early PTB= birth ≤ 34 weeks gestation; late PTB= birth between 35-36 weeks gestation; PTSD=post-traumatic stress disorder; R-CTS=Revised Conflict Tactics Scale short form; RSA=respiratory sinus arrhythmia; SBP= systolic blood pressure; SCID=Structural Clinical Interview for DSM; SCN=admission to special care nursery; SES=socioeconomic status; SFP=Still Face Paradigm; SGA=small for gestational age; SHCN=special health care need; SLE=Stressful life events; SLEI=Lobel and Zambrana Stressful Life Events Inventory; SRI= serotonin reuptake inhibitor; STAXI-2=State-Trait Anger Expression Inventory-2; STD= sexually transmitted disease; STRAIN=Stress and Adversity Inventory; TL=telomere length; VLBW=very low birth weight (< 1500g); VPTB=very preterm birth (<34 weeks gestation)

Note: Brunst et al. (2017) and Sternthal et al. (2011) were produced from the same Asthma Coalition on Community Environment and Social Stress (ACCESS) project. Cheng et al. (2016), Witt et al. (2014a,b), Witt et al. (2015), and Witt et al. (2016) were all produced from the same the Early Childhood Longitudinal Study-Birth Cohort. Cammack et al. (2019), Flagg et al. (2014), Ihongbe (2018), and Strutz et al. (2014) were all produced from the National Longitudinal Study of Adolescent to Adult Health (Add Health)

Supplemental Appendix F. Newcastle Ottawa Scale for Quality Assessment for Cohort Studies Criteria

Selection (5 maximum total points):
**Representativeness of the exposed cohort**<br>*Enter 0 or 1:*<br>1 = truly representative of the average _____ in the community<br>1 = somewhat representative of the average _____ in the community<br>0 = selected group of users (e.g., nurses, volunteers)<br>0 = no description of the derivation of the cohort
**Selection of the non-exposed cohort**<br>*Enter 0 or 1:*<br>1 = drawn from the same community as the exposed cohort<br>0 = drawn from a different source<br>0 = no description of the derivation of the non-exposed cohort
***Ascertainment of exposure**<br>*Enter 0 or 1:*<br>1 = biological test (e.g., blood/urine)<br>1 = structured interview<br>1 = written self-report that characterizes dose (current or cumulative)<br>0 = written self-report without quantification of exposure<br>0 = no description
***Ascertainment of exposure done prospectively or retrospectively**<br>*Enter 0 or 1:*<br>1 = Prospectively<br>0 = Retrospectively
**Demonstration that outcome of interest was not present at start of study, OR baseline assessment**<br>*Enter 0 or 1:*<br>1= yes<br>0 = no
**Comparability (2 maximum total points):**
**Comparability of cohorts on the basis of the design or analysis**<br>*Add points: Minimum 0, Maximum 2*<br>1 = study accounts/controls for ______ (most important factor)<br>1 = study controls for any additional factor<br>0 = no adjustment for potential confounders
**Outcome (3 maximum total points):**
***Assessment of outcome**<br>*Enter 0 or 1:*<br>1 = objective measure<br>1 = validated self-report measures<br>0 = no information or non-validated measures

**Was follow-up long enough for outcomes to occur?**<br>*Enter 0 or 1:*<br>1 = yes (select an adequate follow up period for outcome of interest)<br>0 = no
**Adequacy of follow-up of cohorts**<br>*Enter 0 or 1:*<br>1 = complete follow-up; all subjects accounted for<br>1 = subjects lost to follow-up unlikely to introduce bias - small number lost → ___% (select an adequate %) or description was provided of those lost<br>0 = follow-up rate < ___% (select an adequate %) and no description of those lost<br>0 = no statement

*Modified based on Kansagara et al. (2017) – Benefits and harms of cannabis in chronic pain or post-traumatic stress disorder: A systematic review

Supplemental Appendix G. Newcastle Ottawa Scale for Quality Assessment for Cohort Studies

**CRITERIA CATEGORIES**	Astone et al. (2007)	Brunst et al. (2017)	Cammack et al. (2019)	Flagg et al. (2014)	Freeman (2014)
Representativeness of the exposed cohort	0 – select group of mothers (convenience sampling)	0 – select group of pregnant women (convenience sampling)	1 – truly representative of the average U.S. school (stratified sampling)	1 – truly representative of the average adolescent in the U.S. (probability sampling)	1 – truly representative of the average adolescent in the U.S. (probability sampling)
Selection of the non-exposed cohort	1 – drawn from the same community as the exposed cohort (same sample)	1 – drawn from the same community as the exposed cohort (same sample)	1 – drawn from the same community as the exposed cohort (same sample)	1 – drawn from the same community as the exposed cohort (same sample)	1 – drawn from the same community as the exposed cohort (same sample)
Ascertainment of exposure	1 – structured interview (face-to-face interview)	1 – written self-report that characterizes dose (validated self-report measure [R-CTS])	1 – written self-report that characterizes dose	1 – written self-report that characterizes dose	1 – written self-report that characterizes dose
Ascertainment of exposure done prospectively or retrospectively	1 – prospectively	0 – retrospectively	0 – retrospectively	1 – prospectively	1 – prospectively
Demonstration that outcome of interest was not present at start of study, OR baseline assessment	1 – yes	1 – yes	1 – yes	1 – yes	1 – yes
Comparability of cohorts on the basis of the design or analysis	1 – study controls for any additional factors (e.g., maternal prenatal health, SES, smoking)	1 – study controls for any additional factors (e.g., child sex, maternal age, education, race, sex & birthweight)	1 – study controls for any additional factors (e.g., maternal race & childhood SES)	1 – study controls for any additional factors (e.g., grandparental education, maternal birthweight, age, substance use)	1 – study controls for any additional factors (e.g., maternal health, prenatal environment, current SES)
Assessment of outcome	0 – non-validated measure (maternal report)	1 – objective measure (maternal report of clinician-diagnosed asthma)	0 – non-validated measure (maternal report)	0 – non-validated measure (maternal report)	0 – non-validated measure (maternal report)
Was follow-up long enough for outcomes to occur?	1 – yes (offspring was born)	1 – yes (offspring was born)	1 – yes (offspring was born)	1 – yes (offspring was born)	1 – yes (offspring was born)
Adequacy of follow-up of prospective cohorts/Adequacy of response of	1 – subjects lost to follow-up unlikely to introduce bias (< 3% of offspring lost)	1 – subjects lost to follow-up unlikely to introduce bias (< 4% lost)	1 – subjects lost to follow-up unlikely to introduce bias (< 20% lost)	1 – subjects lost to follow-up unlikely to introduce bias (< 30% lost)	0 – no statement on % of subjects lost to follow-up

retrospective cohorts					
Risk of Bias (ROB):	Moderate ROB; the primary source of bias was the non-representative sample used; maternal report of offspring health was also used	Moderate ROB; the primary source of bias was the non-representative sample used; a retrospective measure of maternal preconception adversity and maternal report of offspring health were also used	Low ROB; the primary source of bias was the retrospective measure of maternal preconception adversity; maternal report of offspring health was also used	Relatively low ROB; the primary source of bias was the retrospective measure of maternal preconception adversity; maternal report of offspring health was also used	Moderate ROB; the primary source of bias was the lack of information about participants' response rate; maternal report of offspring health was also used

**CRITERIA CATEGORIES**	Hillis et al. (2004)	Ihongbe (2018)	Kerkar et al. (2020)	Strutz et al. (2014)	Hilmert et al. (2014)
Representativeness of the exposed cohort	0 – select group of women (convenience sampling)	1 – truly representative of the average adolescent in the U.S. (stratified random sampling)	0 – select group of women (convenience sampling)	1 – truly representative of the average adolescent in the U.S. (probability sampling)	0 – select group of pregnant women (convenience sampling)
Selection of the non-exposed cohort	1 – drawn from the same community as the exposed cohort (same sample)	1 – drawn from the same community as the exposed cohort (same sample)	1 – drawn from the same community as the exposed cohort (same sample)	1 – drawn from the same community as the exposed cohort (same sample)	1 – drawn from the same community as the exposed cohort (same sample)
Ascertainment of exposure	1 – written self-report that characterizes dose (validated self-report measure [ACEs Questionnaire])	1 – written self-report that characterizes dose	1 – written self-report that characterizes dose (validated self-report measure [ACEs Questionnaire])	1 – written self-report that characterizes dose	1 - structured interview
Ascertainment of exposure done prospectively or retrospectively	0 – retrospectively	0 – retrospectively	0 – retrospectively	0 – retrospectively	0 – retrospectively
Demonstration that outcome of interest was not present at start of study, OR baseline assessment	0 – no	1 – yes	0 – no	1 – yes	1 – yes
Comparability of cohorts on the basis of the design or analysis	1 – study controls for any additional factors (e.g., maternal age, race, education, & adolescent pregnancy)	1 – study controls for any additional factors (e.g., maternal age, education, receipt of prenatal care, prenatal substance use)	1 – study controls for any additional factors (e.g., maternal age at pregnancy, BMI, education, smoking)	1 – study controls for any additional factors (e.g., maternal preconception BMI, substance use, marital status)	1 – study controls for any additional factors (e.g., maternal BMI, SES, exposure to SLEs)
Assessment of outcome	0 – non-validated measure (maternal report)	0 – non-validated measure (maternal report)	0 – non-validated measure (maternal report)	0 – non-validated measure (maternal report)	1 - objective measure (medical records)
Was follow-up long enough for outcomes to occur?	1 – yes (offspring was born)	1 – yes (offspring was born)	1 – yes (offspring was born)	1 – yes (offspring was born)	1 – yes (offspring was born)
Adequacy of follow-up of prospective cohorts/Adequacy of response of	0 – response rate < 70% (68%)	1 – subjects lost to follow-up unlikely to introduce bias (< 30% lost at each wave of	0 – no statement on % of non-respondents	1 – subjects lost to follow-up unlikely to introduce bias (< 30% lost at each wave of data	1 – subjects lost to follow-up unlikely to introduce bias (~7% lost)

retrospective cohorts		data collection)		collection)	
Risk of Bias (ROB):	High ROB; the primary sources of bias were the non-representative sample used and the inadequate participant response rate obtained; a retrospective measure of maternal preconception adversity and maternal report of offspring health were also used	Low ROB; the primary source of bias was the retrospective measure of maternal preconception adversity; maternal report of offspring health was also used	High ROB; the primary sources of bias were the lack of information about participants' response rate and the non-representative sample used; a retrospective measure of maternal preconception adversity and maternal report of offspring health were also used	Low ROB; the primary source of bias was the retrospective measure of maternal preconception adversity; maternal report of offspring health was also used	Moderate ROB; the primary source of bias was the non-representative sample used; a retrospective measure of maternal preconception adversity was also used

**CRITERIA CATEGORIES**	Sealy-Jefferson et al. (2019)	Dominguez et al. (2008)	Gray et al. (2017)	Margerison-Zilko et al. (2017)	Masho et al. (2015)
Representativeness of the exposed cohort	0 – select group of women (convenience sampling)	0 – select group of pregnant women (convenience sampling)	0 – select group of pregnant women (convenience sampling)	0 – select group of pregnant women (convenience sampling)	0 – select group of pregnant women (convenience sampling)
Selection of the non-exposed cohort	1 – drawn from the same community as the exposed cohort (same sample)	1 – drawn from the same community as the exposed cohort (same sample)	1 – drawn from the same community as the exposed cohort (same sample)	1 – drawn from the same community as the exposed cohort (same sample)	1 – drawn from the same community as the exposed cohort (same sample)
Ascertainment of exposure	1 – written self-report that characterizes dose (validated self-report early-life neighborhood social control & social disorder scales)	1 – structured interview	1 – written self-report that characterizes dose (validated self-report measure [ACEs Questionnaire])	1 – structured interview (detailed in-person & self-recorded interview)	1 – written self-report that characterizes dose (validated self-report measures [SLEI & PSS])
Ascertainment of exposure done prospectively or retrospectively	0 – retrospectively	0 – retrospectively	0 – retrospectively	0 – retrospectively	0 – retrospectively
Demonstration that outcome of interest was not present at start of study, OR baseline assessment	0 – no	1 – yes	1 – yes	1 – yes	1 – yes
Comparability of cohorts on the basis of the design or analysis	1 – study controls for any additional factors (e.g., maternal age, marital status, educational attainment, income)	1 – study controls for any additional factors (e.g., maternal medical & sociodemographic risk factors, gestational age at delivery, spontaneous labor)	1 – study controls for any additional factors (e.g., gestational age, maternal education, infant sex)	1 – study controls for any additional factors (e.g., maternal education, parity, marital status)	1 – study controls for any additional factors (e.g., maternal age, education, adequacy of prenatal care)
Assessment of outcome	1 - objective measure (medical records)	1 - objective measure (medical records)	1 - objective measure (EEG)	1 - objective measure (medical records)	1 - objective measure (medical records)
Was follow-up long enough for outcomes to occur?	1 – yes (offspring was born)	1 – yes (offspring was born)	1 – yes (offspring was born)	1 – yes (offspring was born)	1 – yes (offspring was born)
Adequacy of follow-up of prospective cohorts/Adequacy of response of	1 – non-respondents unlikely to introduce bias (29% of participants	1 – subjects lost to follow-up unlikely to introduce bias (< 30% lost)	0 – no statement on % of subjects lost to follow-up	1 – subjects lost to follow-up unlikely to introduce bias (< 1% lost)	0 – no statement on % of subjects lost to follow-up

retrospective cohorts	approached declined participation)				
Risk of Bias (ROB):	Moderate ROB; the primary source of bias was the non-representative sample used; a retrospective measure of maternal preconception adversity was also used	Moderate ROB; the primary source of bias was the non-representative sample used; a retrospective measure of maternal preconception adversity was also used	High ROB; the primary sources of bias were lack of information about participants' follow-up rate and the non-representative sample used; a retrospective measure of maternal preconception adversity was also used	Moderate ROB; the primary source of bias was the non-representative sample used; a retrospective measure of maternal preconception adversity was also used	High ROB; the primary sources of bias were the lack of information about participants' follow-up rate and the non-representative sample used; a retrospective measure of maternal preconception adversity was also used

**CRITERIA CATEGORIES**	Seng et al. (2011)	Blackmore et al. (2016)	Cheng et al. (2016)	Mersky & Lee (2019)	Noll et al. (2007)
Representativeness of the exposed cohort	0 – select group of pregnant women (convenience sampling)	0 – select group of pregnant women (convenience sampling)	1 – truly representative of the average child born in the U.S. (probability sampling)	0 – select group of women (convenience sampling)	0 – select group of women (convenience sampling)
Selection of the non-exposed cohort	1 – drawn from the same community as the exposed cohort (same sample)	1 – drawn from the same community as the exposed cohort	1 – drawn from the same community as the exposed cohort (same sample)	1 – drawn from the same community as the exposed cohort (same sample)	1 – drawn from the same community as the exposed cohort
Ascertainment of exposure	1 – written self-report that characterizes dose (validated self-report measure [Life Stressor Checklist])	1 – written self-report that characterizes dose (validated self-report measure [PTSD section of the SCID])	1 – structured interview	1 – written self-report that characterizes dose (validated self-report measure [ACEs Questionnaire])	1 – structured interview (referral by CPS agencies)
Ascertainment of exposure done prospectively or retrospectively	0 – retrospectively	0 – retrospectively	0 – retrospectively	0 – retrospectively	1 – prospectively
Demonstration that outcome of interest was not present at start of study, OR baseline assessment	1 – yes	1 – yes	0 – no	0 – no	1 – yes
Comparability of cohorts on the basis of the design or analysis	1 – study controls for any additional factors (e.g., maternal poverty, chronic condition, antepartum complication, substance use, adequate prenatal care)	1 – study controls for any additional factors (e.g., maternal age, BMI, prenatal substance use)	1 – study controls for any additional factors (e.g., maternal chronic conditions, parity, age, SES)	1 – study controls for any additional factors (e.g., maternal age, race/ethnicity, and education)	1 – study controls for any additional factors (e.g., maternal minority status, offspring number of siblings in sample)
Assessment of outcome	1 - objective measure (medical records)	1 - objective measure (medical records)	1 - objective measure (birth certificate)	1 - objective measure (archival program records)	1 - objective measure (hospital records)
Was follow-up long enough for outcomes to occur?	1 – yes (offspring was born)	1 – yes (offspring was born)	1 – yes (offspring was born)	1 – yes (offspring was born)	1 – yes (offspring was born)
Adequacy of follow-up of prospective cohorts/Adequacy of response of	0 – follow-up rate < 70% (~53%)	1 – subjects lost to follow-up unlikely to introduce bias (< 5% lost)	0 – no statement on % of non-respondents	0 – no statement on % of non-respondents	1 – subjects lost to follow-up unlikely to introduce bias (~4% lost)

retrospective cohorts					
Risk of Bias (ROB):	High ROB; the primary sources of bias were the non-representative sample used and the inadequate % of participants retained at follow up; a retrospective measure of maternal preconception adversity was also used	Moderate ROB; the primary source of bias was the non-representative sample used; a retrospective measure of maternal preconception adversity was also used	Low ROB; the primary source of bias was the retrospective measure of maternal preconception adversity used	High ROB; the primary sources of bias were the lack of information about participants' response rate and the non-representative sample used; a retrospective measure of maternal preconception adversity was also used	Moderate ROB; the primary source of bias was the non-representative sample used

**CRITERIA CATEGORIES**	Smith et al. (2016)	Sternthal et al. (2011)	Witt et al. (2014a)	Witt et al. (2014b)	Witt et al. (2015)
Representativeness of the exposed cohort	0 – select group of pregnant women (convenience sampling)	0 – select group of pregnant women (convenience sampling)	1 – truly representative of the average child born in the U.S. (probability sampling)	1 – truly representative of the average child born in the U.S. (probability sampling)	1 – truly representative of the average child born in the U.S. (probability sampling)
Selection of the non-exposed cohort	1 – drawn from the same community as the exposed cohort (same sample)	1 – drawn from the same community as the exposed cohort (same sample)	1 – drawn from the same community as the exposed cohort (same sample)	1 – drawn from the same community as the exposed cohort (same sample)	1 – drawn from the same community as the exposed cohort (same sample)
Ascertainment of exposure	1 – written self-report that characterizes dose (modified validated self-report measure [ETI-SF])	1 – written self-report that characterizes dose (self-report of binary measure)	1 – structured interview	1 – structured interview	1 – structured interview
Ascertainment of exposure done prospectively or retrospectively	0 – retrospectively	0 – retrospectively	0 – retrospectively	0 – retrospectively	0 – retrospectively
Demonstration that outcome of interest was not present at start of study, OR baseline assessment	1 – yes	1 – yes	0 – no	0 – no	0 – no
Comparability of cohorts on the basis of the design or analysis	1 – study controls for any additional factors (e.g., maternal race/ethnicity [other additional factors were mediators (e.g., maternal smoking, education, marital status)])	1 – study controls for any additional factors (e.g., maternal atopy, nativity status, race/ethnicity, child sex)	2 – study controls for most important factor (prenatal adversity) and any additional factors (e.g., maternal sociodemographic & health factors, prenatal stress)	2 – study controls for most important factor (prenatal adversity) and any additional factors (e.g., maternal sociodemographic & health factors, prenatal stress)	2 – study controls for most important factor (prenatal adversity) and any additional factors (e.g., maternal sociodemographic & health factors, prenatal stress)
Assessment of outcome	1 - objective measure (medical records)	1 - objective measure (enzyme immunoassay)	1 - objective measure (birth certificate)	1 - objective measure (birth certificate)	1 - objective measure (birth certificate)
Was follow-up long enough for outcomes to occur?	1 – yes (offspring was born)	1 – yes (offspring was born)	1 – yes (offspring was born)	1 – yes (offspring was born)	1 – yes (offspring was born)
Adequacy of follow-up of prospective cohorts/Adequacy of response of	1 – subjects lost to follow-up unlikely to introduce bias (~14% lost)	1 – subjects lost to follow-up unlikely to introduce bias (~23% lost)	1 – non-respondents unlikely to introduce bias (< 24% of participants approached	1 – non-respondents unlikely to introduce bias (< 24% of participants approached declined	0 – no statement on % of non-respondents

retrospective cohorts			declined participation)	participation)	
Risk of Bias (ROB):	Moderate ROB; the primary source of bias was the non-representative sample used; a retrospective measure of maternal preconception adversity was also used	Moderate ROB; the primary source of bias was the non-representative sample used; a retrospective measure of maternal preconception adversity was also used	Low ROB; the primary source of bias was the retrospective measure of maternal preconception adversity used	Low ROB; the primary source of bias was the retrospective measure of maternal preconception adversity used	Low ROB; the primary source of bias was the retrospective measure of maternal preconception adversity used

**CRITERIA CATEGORIES**	Witt et al. (2016)	Cowell et al. (2021)	Gavin et al. (2011)	Gillespie et al. (2017)	Jones et al. (2019)
Representativeness of the exposed cohort	1 – truly representative of the average child born in the U.S. (probability sampling)	0 – select group of pregnant women (convenience sampling)	0 – select group of elementary school children (convenience sampling)	0 – select group of pregnant women (convenience sampling)	0 – select group of pregnant women (convenience sampling)
Selection of the non-exposed cohort	1 – drawn from the same community as the exposed cohort (same sample)	1 – drawn from the same community as the exposed cohort (same sample)	1 – drawn from the same community as the exposed cohort (same sample)	1 – drawn from the same community as the exposed cohort (same sample)	1 – drawn from the same community as the exposed cohort (same sample)
Ascertainment of exposure	1 – structured interview	1 – written self-report that characterizes dose (validated self-report measure [CTQ])	1 – written self-report that characterizes dose (validated self-report measure [CTQ])	1 – written self-report that characterizes dose (validated self-report measure [STRAIN])	1 – written self-report that characterizes dose (validated self-report measure [ACEs Questionnaire])
Ascertainment of exposure done prospectively or retrospectively	0 – retrospectively	0 – retrospectively	0 – retrospectively	0 – retrospectively	0 – retrospectively
Demonstration that outcome of interest was not present at start of study, OR baseline assessment	0 – no	1 – yes	1 – yes	1 – yes	1 – yes
Comparability of cohorts on the basis of the design or analysis	2 – study controls for most important factor (prenatal adversity) and any additional factors (e.g., maternal sociodemographic & health factors)	1 – study controls for any additional factors (e.g., maternal age, parity, education, smoking)	1 – study controls for any additional factors (e.g., maternal BMI, prenatal substance use)	1 – study controls for any additional factors (e.g., maternal adulthood stress, sleep quality, hours awake prior to venipuncture)	1 – study controls for any additional factors (e.g., infant sex, maternal race, prenatal smoking)
Assessment of outcome	1 - objective measure (birth certificate)	1 – objective measure (medical records)	0 – non-validated measure (maternal report)	1 – objective measure (prenatal and labor & delivery records)	1 – objective measure (ECG, placental TL)
Was follow-up long enough for outcomes to occur?	1 – yes (offspring was born)	1 – yes (offspring was born)	1 – yes (offspring was born)	1 – yes (offspring was born)	1 – yes (offspring was born)
Adequacy of follow-up of prospective cohorts/Adequacy of response of	1 – non-respondents unlikely to introduce bias (< 24% of participants approached	1 – subjects lost to follow-up unlikely to introduce bias (< 30% lost)	1 – description of subjects lost was provided (no differences between subjects retained	1 – subjects lost to follow-up unlikely to introduce bias (1% lost)	0 – no statement on % of subjects lost to follow-up

retrospective cohorts	declined participation)		and lost)		
Risk of Bias (ROB):	Low ROB; the primary source of bias was the retrospective measure of maternal preconception adversity used	Moderate ROB; the primary source of bias was the non-representative sample used; a retrospective measure of maternal preconception adversity was also used	Moderate ROB; the primary source of bias was the non-representative sample used; a retrospective measure of maternal preconception adversity and maternal report of offspring health were also used	Moderate ROB; the primary source of bias was the non-representative sample used; a retrospective measure of maternal preconception adversity was also used	High ROB; the primary sources of bias were the lack of information about participants' follow-up rate and the non-representative sample used; a retrospective measure of maternal preconception adversity was also used

**CRITERIA CATEGORIES**	Miller et al. (2017)
Representativeness of the exposed cohort	0 – select group of pregnant women (convenience sampling)
Selection of the non-exposed cohort	1 – drawn from the same community as the exposed cohort (same sample)
Ascertainment of exposure	1 – written self-report that characterizes dose
Ascertainment of exposure done prospectively or retrospectively	0 – retrospectively
Demonstration that outcome of interest was not present at start of study, OR baseline assessment	1 – yes
Comparability of cohorts on the basis of the design or analysis	1 – study controls for any additional factors (e.g., maternal demographics, education, and obstetrical confounders [e.g., nulliparity])
Assessment of outcome	1 - objective measure (maternal and neonatal charts)
Was follow-up long enough for outcomes to occur?	1 – yes (offspring was born)
Adequacy of follow-up of prospective cohorts/Adequacy of response of retrospective cohorts	1 – subjects lost to follow-up unlikely to introduce bias (< 30% lost)
Risk of Bias (ROB):	Moderate ROB; the primary source of bias was the non-representative sample used; a retrospective measure of maternal preconception adversity was also used

Supplemental Appendix H. Newcastle Ottawa Scale for Quality Assessment for Cross-Sectional Studies Criteria

**Selection (5 maximum total points):**
**Representativeness of the sample**<br>*Enter 0 or 1:*<br>1 = truly representative of the average in the target population (all subjects or random sampling)<br>1 = somewhat representative of the average in the target population (non-random sampling)<br>0 = select group of users (e.g., nurses, volunteers)<br>0 = no description of the sampling strategy
**Non-respondents**<br>*Enter 0 or 1:*<br>1 = comparability between respondents and non-respondents characteristics is established and the response rate is satisfactory<br>0 = the response rate is unsatisfactory, or the comparability between respondents and non-respondents is unsatisfactory<br>0 = no description of the response rate or the characteristics of the respondents and non-respondents
**Sample size**<br>*Enter 0 or 1:*<br>1 = justified and satisfactory<br>0 = not justified
**Ascertainment of exposure**<br>*Enter 0 or 1:*<br>1 = validated measurement tool<br>1 = non-validated measurement tool that is available or described<br>0 = no description of the measurement tool
***Ascertainment of exposure done prospectively or retrospectively**<br>*Enter 0 or 1:*<br>1 = Prospectively<br>0 = Retrospectively
**Comparability (2 maximum total points):**
**The subjects in different outcome groups are comparable, based on the study design or analysis - confounding factors are controlled**<br>*Add points: Minimum 0, Maximum 2*<br>1 = study accounts/controls for the most important factor (select one)<br>1 = study controls for any additional factor<br>0 = no adjustment for potential confounders

**Outcome (3 maximum total points):**
**Assessment of outcome**<br>*Enter 0 or 1:*<br>2 = independent blind assessment<br>1 = record linkage<br>1 = self-report<br>0 = no description
**Statistical test**<br>*Enter 0 or 1:*<br>1 = the statistical test used to analyze the data is clearly described and appropriate, and the measurement of the association is presented, including confidence intervals and the probability level (p value)<br>0 = the statistical test is not appropriate, not descried or incomplete

*Adapted for cross-sectional studies by Herzog et al. (2013) - Are healthcare workers' intentions to vaccinate related to their knowledge, beliefs and attitudes? A systematic review

Supplemental Appendix I. Newcastle Ottawa Scale for Quality Assessment for Cross-Sectional Studies

**CRITERIA CATEGORIES**	Jovanovic et al. (2011)	Rowell (2020)	Chen et al. (2017)	Daniels et al. (2020)	Stein et al. (2000)
Representativeness of the exposed cohort	0 – select group of children (convenience sampling)	0 – select group of pregnant women (convenience sampling)	0 – select group of children (convenience sampling)	0 – select group of women (convenience sampling)	1 - somewhat representative of the average homeless woman in LA (stratified sampling)
Non-respondents	0 – no description of the response rate	0 – no description of the response rate	1 – the response rate is satisfactory (~76%)	0 – no description of the response rate	1 - the response rate is satisfactory (81%)
Sample size	0 – not satisfactory (36 children)	0 – not satisfactory (31 pregnant women)	1 – satisfactory (150 children)	1 – satisfactory (208 women)	1 - (237 homeless women with live births in the last 3 years)
Ascertainment of exposure	1 – validated measurement tool (self-reported CTQ)	1 – validated measurement tool (self-reported ACEs Questionnaire)	1 – non-validated measurement tool that is available or described (self-reported childhood home ownership)	1 – non-validated measurement tool that is available or described (adolescent and childhood exposure to direct & vicarious racial discrimination)	1 - non-validated measurement tool that is available or described (self-reported yes or no to rape or sexual abuse and assault before age 18)
Ascertainment of	0 – retrospectively	0 – retrospectively	0 – retrospectively	0 – retrospectively	0 – retrospectively

exposure done prospectively or retrospectively					
Comparability: The subjects in different outcome groups are comparable, based on the study design or analysis - confounding factors are controlled	1 – study controls for any additional factors (e.g., child trauma exposure, child sex & age, maternal PTSD & depression)	1 – study controls for any additional factors (e.g., maternal distress, trimester, BMI, systolic & diastolic blood pressure)	1 – study controls for any additional factors (e.g., child age, sex, ethnicity use of beta agonists, use of inhaled corticosteroids)	1 – study controls for any additional factors (e.g., maternal parity, household income, educational attainment, employment status, marital status)	1 – study controls for any additional factors (e.g., maternal age, nulliparity, antenatal complications)
Assessment of outcome	2 – independent or blind assessment (EMG & ECG)	2 – independent or blind assessment (doulas present at delivery)	2 – independent or blind assessment (blood samples)	1 – self-report (maternal report)	1 – self-report (maternal report)
Statistical test	1 - the statistical test used is clearly described and appropriate & the measurement of the association is presented (ANOVAs & hierarchical regressions, coefficients & F statistics, $p < .05$)	1 - the statistical test used is clearly described and appropriate & the measurement of the association is presented (linear regressions, coefficients, $p < .05$)	1 - the statistical test used is clearly described and appropriate & the measurement of the association is presented (ANCOVAs & multiple regressions, coefficients & F statistics, 95% CIs, $p < .05$)	1 - the statistical test used is clearly described and appropriate & the measurement of the association is presented (logistic regression, ORs, 95% CIs, $p < .05$)	1 - the statistical test used is clearly described and appropriate & the measurement of the association is presented (SEM, coefficients, $p < .05$)
Risk of Bias (ROB):	High ROB; the primary sources of bias were the small and non-representative sample used, and lack of information about participants' response rate; a retrospective measure of maternal preconception adversity was also used	High ROB; the primary sources of bias were the small and non-representative sample used, and lack of information about participants' response rate; a retrospective measure of maternal preconception adversity was also used	Moderate ROB; the primary source of bias was the non-representative sample used; a retrospective measure of maternal preconception adversity was also used	High ROB; the primary sources of bias were the non-representative sample used and the lack of information about participants' response rate; a retrospective measure of maternal preconception adversity and maternal report of offspring health were also used	Low ROB; the primary source of bias was the retrospective measure of maternal preconception adversity; maternal report of offspring health was also used

**CRITERIA CATEGORIES**	Lê-Scherban et al. (2018)
Representativeness of the exposed cohort	1 - somewhat representative of the average resident of Philadelphia & its surrounding counties (stratified sampling)
Non-respondents	0 – the response rate is unsatisfactory (67%)
Sample size	1 – satisfactory (350 parents & their children)
Ascertainment of exposure	1 - validated measurement tool (self-reported adapted ACEs Questionnaire & BRFSS ACE module)
Ascertainment of exposure done prospectively or retrospectively	0 – retrospectively
Comparability:<br>The subjects in different outcome groups are comparable, based on the study design or analysis - confounding factors are controlled	1 – study controls for any additional factors (e.g., parent age & sex, child age & sex)
Assessment of outcome	1 – self-report (parent report)
Statistical test	1 - the statistical test used is clearly described and appropriate & the measurement of the association is presented (logistic regression, ORs, 95% CIs, $p < .05$)
Risk of Bias (ROB):	Moderate ROB; the primary source of bias was the inadequate participant response rate obtained; a retrospective measure of maternal preconception adversity and maternal report of offspring health were also used

Supplemental Appendix J. Newcastle Ottawa Scale for Quality Assessment for Case-Control Studies Criteria

**Selection (A study can be awarded a maximum of one star for each numbered item):**
**1) Is the case definition adequate? Representativeness of the exposed cohort**<br>a) yes, with independent validation*<br>b) yes, e.g., record linkage or based on self-reports<br>c) no description
**2) Representativeness of the cases**

a) consecutive or obviously representative series of cases*<br>b) potential for selection biases or not stated
**3) Selection of controls**<br>a) community controls*<br>b) hospital controls<br>c) no description
**4) Definition of controls**<br>a) no history of disease (endpoint)*<br>b) no description of source
**Comparability (A study can be awarded a maximum of one star):**
**1) Comparability of cases and controls on the basis of the design or analysis**<br>a) study controls for **________________** (Select the most important factor.)*<br>b) study controls for any additional factor* (These criteria could be modified to indicate specific control for a second important factor.)
**Exposure (A study can be awarded a maximum of one star for each numbered item):**
**1) Ascertainment of exposure**<br>a) secure record (e.g., surgical records)*<br>b) structured interview where blind to case/control status*<br>c) interview not blinded to case/control status<br>d) written self-report or medical record only<br>e) no description
**2) Same method of ascertainment for cases and controls**<br>a) yes*<br>b) no
**3) Non-Response rate**<br>a) same rate for both groups*<br>b) non respondents described<br>c) rate different and no designation

Supplemental Appendix K. Newcastle Ottawa Scale for Quality Assessment for Case-Control Studies

**CRITERIA CATEGORIES**	Freedman et al. (2017)
Is the case definition adequate?	1 – yes, with independent validation (medical records)
Representativeness of the cases	1 – consecutive or obviously representative series (population-based study with stratified random sampling)

Selection of controls	1 – community controls (same birth hospitals as cases)
Definition of controls	1 – index delivery did not result in stillbirth
Comparability of cases and controls on the basis of the design or analysis	1 – study controls for any additional factors (e.g., maternal education, age, time between index delivery & follow-up interview)
Ascertainment of exposure	0 – written self-report (CTQ)
Same method of ascertainment for cases and controls	1 – yes (CTQ)
Non-response rate	0 – rate different for each group (17% non-response for cases vs. 25% non-response for controls)
Risk of bias (ROB):	Moderate ROB; the primary source of bias was the different participant response rates in the case and control groups; a retrospective measure of maternal preconception adversity was also used
